# Secondary Placental Defects in *Cxadr* Mutant Mice

**DOI:** 10.3389/fphys.2019.00622

**Published:** 2019-05-29

**Authors:** Jennifer E. Outhwaite, Jatin Patel, David G. Simmons

**Affiliations:** ^1^ Faculty of Medicine, School of Biomedical Sciences, The University of Queensland, Brisbane, QLD, Australia; ^2^ Translational Research Institute, UQ Diamantina Institute, The University of Queensland, Brisbane, QLD, Australia

**Keywords:** placenta, *Cxadr*, fetal heart, fetal circulation, placenta-heart axis

## Abstract

The Coxsackie virus and adenovirus receptor (CXADR) is an adhesion molecule known for its role in virus-cell interactions, epithelial integrity, and organogenesis. Loss of *Cxadr* causes numerous embryonic defects in mice, notably abnormal development of the cardiovascular system, and embryonic lethality. While CXADR expression has been reported in the placenta, the precise cellular localization and function within this tissue are unknown. Since impairments in placental development and function can cause secondary cardiovascular abnormalities, a phenomenon referred to as the placenta-heart axis, it is possible placental phenotypes in *Cxadr* mutant embryos may underlie the reported cardiovascular defects and embryonic lethality. In the current study, we determine the cellular localization of placental *Cxadr* expression and whether there are placental abnormalities in the absence of *Cxadr*. In the placenta, CXADR is expressed specifically by trophoblast labyrinth progenitors as well as cells of the visceral yolk sac (YS). In the absence of *Cxadr*, we observed altered expression of angiogenic factors coupled with poor expansion of trophoblast and fetal endothelial cell subpopulations, plus diminished placental transport. Unexpectedly, preserving endogenous trophoblast *Cxadr* expression revealed the placental defects to be secondary to primary embryonic and/or YS phenotypes. Moreover, further tissue-restricted deletions of *Cxadr* suggest that the secondary placental defects are likely influenced by embryonic lineages such as the fetal endothelium or those within the extraembryonic YS vascular plexus.

## Introduction

The Coxsackie virus and adenovirus receptor (CXADR) was originally characterized as a viral receptor that enables both Coxsackie B virus and adenovirus serotypes to gain entry into host cells (Bergelson et al., 1997; Tomko et al., 1997). Since then, CXADR has been recognized as a cell adhesion molecule, a component of apical junction complexes, and has been implicated in numerous physiological functions during embryonic development and epithelial cell interactions (Supplementary Table S1). What is clear from the literature is that CXADR is variably expressed during development, is promiscuous in its interactions, and is required by numerous tissues during organogenesis, with its roles in development differing to those in adult tissues (Supplementary Tables S1, S2). In particular, CXADR has been implicated in cell-cell junction integrity during cardiac and lymphatic development, as well as facilitating a permeability barrier in the trophectoderm of pre-implantation blastocysts (Asher et al., 2005; Dorner et al., 2005; Chen et al., 2006; Mirza et al., 2012; Krivega et al., 2014; Kwon et al., 2016; Oh et al., 2016).

Ubiquitous deletion of *Cxadr* is embryonic lethal between embryonic day (E) 11.5 and E13.5 with some null embryos variably reported to exhibit edema, hemorrhage, delayed heart development or valve formation, distended pericardia, enlarged aorta and cardinal veins, increased cardiomyocyte apoptosis, or proliferation (Asher et al., 2005; Dorner et al., 2005; Chen et al., 2006). Studies have therefore largely concentrated on the cardiac defects that are present when functional CXADR is lost and suggest that these defects underlie the embryonic lethality. However, the described cardiac phenotypes are inconsistent between studies and could, at this developmental stage, be attributed to broader vascular or placental defects, neither of which has been fully addressed. In fact, early cardiac defects associated with placental malformation have been shown to include smaller cardiac chambers, myocardial thinning, pericardial effusion, hemorrhage, increased cardiomyocyte apoptosis, ventricular septal defects, and missing atrioventricular cushions, some of which mirror those reported in the various *Cxadr* null studies (Barak et al., 1999; Schorpp-Kistner et al., 1999a; Adams et al., 2000; Mudgett et al., 2000; Schreiber et al., 2000; Hatano et al., 2003; Galabova-Kovacs et al., 2006; Raffel et al., 2008; Ouseph et al., 2012; Maruyama et al., 2016; Perez-Garcia et al., 2018).

Importantly, placental and heart development are known to be interdependent; a phenomenon coined the placenta-heart axis, previously posited by Barak et al. (1999), which states the occurrence of a primary placental phenotype can result in secondary damage to the developing heart (Barak et al., 1999; Hemberger and Cross, 2001). This theory is supported in the mouse by studies that reveal cardiovascular defects can arise following placental-specific gene deletions; or alternatively, the amelioration of heart malformations follows the restoration of gene expression within the placenta alone: *Pparγ* (Barak et al., 1999), *Fra1* (Schreiber et al., 2000), *Erk2* (Hatano et al., 2003), *p38α Mapk* (Adams et al., 2000; Mudgett et al., 2000), *B-Raf* (Galabova-Kovacs et al., 2006), *JunB* (Schorpp-Kistner et al., 1999a), *Ly6e* (Langford et al., 2018), *Ott1* (Raffel et al., 2008), *Senp2* (Maruyama et al., 2016), *Rb* (Wu et al., 2003; Wenzel et al., 2007), *c-Myc* (Dubois et al., 2008), and *E2f7/E2f8* (Ouseph et al., 2012).

One of the first *Cxadr* knockout studies noted CXADR expression in trophoblast cells of the placenta and described possible altered labyrinth fetal vessel growth at a time consistent with lethality of the embryo. However, it was concluded that the phenotype was mild and would likely not impact development or growth of the embryo (Dorner et al., 2005). The critical bidirectional dialogue between trophoblast epithelium and extraembryonic mesoderm, including fetal endothelium, in driving placental morphogenesis (Lu et al., 2013) is often underappreciated, and the full milieu of required molecular signals has not been fully elucidated. Given expression of CXADR in placenta, the cardiovascular phenotypes and embryonic lethality in *Cxadr* null embryos, and the link between placental impairments and cardiac development, we hypothesized that trophoblast CXADR expression may be required to direct labyrinth morphogenesis, and subsequent impairments may contribute to the lethality of *Cxadr* mutant embryos.

In the current study, we found *Cxadr* is expressed by trophoblast cells of the chorion and is retained in specialized labyrinth trophoblast progenitor cells at the chorioallantoic border; cells known to be critical for labyrinth development, guiding fetal blood vessels and contributing to the trophoblast component of the interhaemal membrane (IHM; Ueno et al., 2013; Walentin et al., 2016). In addition, close examination of the placentas of *Cxadr* mutant embryos found, contrary to previous reports, a severe phenotype comprising diminished labyrinth formation, reduced branching morphogenesis of the IHM, altered trophoblast development (syncytiotrophoblast layer II, SynT-II), inadequate expansion of endovascular progenitor (EVP) cells, and impaired placental transport. Opposing our initial hypothesis, a trophoblast sparing Cre transgene revealed that the severe labyrinth deficits in *Cxadr* null animals are directed by extraplacental phenotypes, and not the trophoblast compartment of the placenta. Loss of extraplacental CXADR results in altered placental expression of angiogenic factors and poor expansion of trophoblast and endothelial cell subpopulations at the chorioallantoic interface. Importantly, while the trophoblast defects are secondary to extraplacental phenotypes, the resulting impact to placental function nevertheless suggests that placental insufficiency may be a major contributing factor to embryonic lethality and the severity of the *Cxadr* null phenotypes, including potentially previously observed heart defects. Moreover, further tissue-restricted deletions of *Cxadr* raise the possibility that the secondary placental defects are likely influenced by embryonic lineages such as the fetal endothelium or those within the extraembryonic YS vascular plexus.

## Materials and Methods

### Mice

Animal experiments conducted in this study were approved by the University of Queensland Animal Ethics Committee and conformed to their guidelines. Toe and ear notch samples were used for genotyping and sequencing of genomic DNA. Genotyping primers are listed in Supplementary Table S3, and details pertaining to the individual mouse lines are outlined in Supplementary Table S4.

Recognition of seminal plug was designated E0.5. All tissues were dissected in cold PBS and fixed in 2 or 4% paraformaldehyde at 4°C overnight. Samples were processed through an ethanol gradient to xylene before being paraffin embedded. Frozen samples were processed through a sucrose gradient before being frozen in OCT-media (Tissue Tek). Alternatively, some tissues were fresh frozen in OCT-media and post-fixed in acetone or methanol following cryosectioning, depending on antibody requirements. 5 and 7 μm paraffin sections and 10 μm OCT sections were used.

### Stereology

Total placental and placental zone volumes were calculated by employing Cavalieri’s principal following processing and sectioning, as previously described (Coan, 2004). Morphometry of fetal and maternal blood spaces was established by randomly selecting several fields of view (3 fields per section/5 sections per placenta, 70 μm apart/minimum of *n* = 5 per genotype) at 40× magnification from across the labyrinth (E10.5 and E11.5). Estimates of volume and absolute surface area were calculated using the test point and test grid cycloid arc counting systems as outlined by Coan, 2004.

### Rhodamine 123

Two hours prior to dissection, Rhodamine 123 (Sigma-Aldrich) was injected subcutaneously (1 μg/g of body weight) into pregnant mice (E10.5, *n* = 3 litters and E11.5, *n* = 3 litters). Embryos and placentas were dissected in cold PBS and imaged directly on a Leica M205FA stereo fluorescent microscope using Leica software. Samples were taken from each embryo for subsequent genotyping. ImageJ software was used to determine fluorescent intensity in the embryos. Data from each profile were then imported into GraphPad Prism 6 and pooled according to genotype (E10.5: +/+ *n* = 7, +/− *n* = 9, −/− *n* = 5; E11.5: +/+ *n* = 6, +/− *n* = 6, −/− *n* = 5). Averaged fluorescent intensities were compared between genotypes and graphed (± SEM).

### Flow Cytometry

Dissociated single cells in PBS/BSA/% EDTA were incubated with various antibody combinations for multiparameter flow acquisition and analysis, as previously described (Patel et al., 2017). The following combinations of antibodies were used to assess endothelial progenitor cell populations: Rat anti-mouse VE-Cadherin FITC, VEGFR2 PE, CD31 PE-Cy7, CD34 Alexa647, and CD45 V450 (Becton Dickinson).

### Real-Time PCR

RNA was extracted using RNAzol BD (Astral Scientific) according to the manufacturer’s instructions. Total RNA was used to generate cDNA (final concentration 100 *n*g/μL; Qiagen Quantitect cDNA synthesis kit), and mRNA expression levels were assessed by iTaq Universal SYBR Green Super Mix (BIO-RAD) as per manufacturer’s instructions. Pre-designed and verified gene-specific primers were purchased from Sigma-Aldrich (KiCqStart qPCR primers; Supplementary Table S5).

### *In situ* Hybridization

The various *in situ* probes were generated with gene-specific primer sets that contained T7 RNA polymerase promoter sequence (TAATACGACTCACTATAGGG) attached to each reverse primer and T3 RNA polymerase promoter sequence (AATTAACCCTCACTAAAGGG) attached to each forward primer. Gene-specific primer sequences are listed in Supplemental Table S6. Sections were processed and stained as previously described (Simmons et al., 2008).

### Immunofluorescence

Immunofluorescence was performed on paraffin and frozen sections. Concentrations and optimization conditions for the various antibodies are listed in Supplementary Table S7. Sections were blocked with 1 × PBS/0.01% Tween 20/5% serum for 1 h at RT. Incubating slides in 0.1% Sudan Black B/70% EtOH for 20 min at RT were performed to reduce auto-fluorescence of red blood cells. Following this, slides were incubated in primary antibody overnight at 4°C, followed by secondary antibody incubation for 1 h at RT (Supplementary Table S7).

### Statistics

Results are represented as mean with variance displayed as standard error of the mean (± SEM). Differences were analyzed using Student’s *t* test with Welsh’s *t*-test correction performed when samples showed unequal variance. *p* < 0.05 was considered significant. Statistical analysis was performed using Prism 6 (GraphPad Software).

## Results

### CXADR Is Expressed in the Mouse Placenta and Yolk Sac

**Figure 1:**
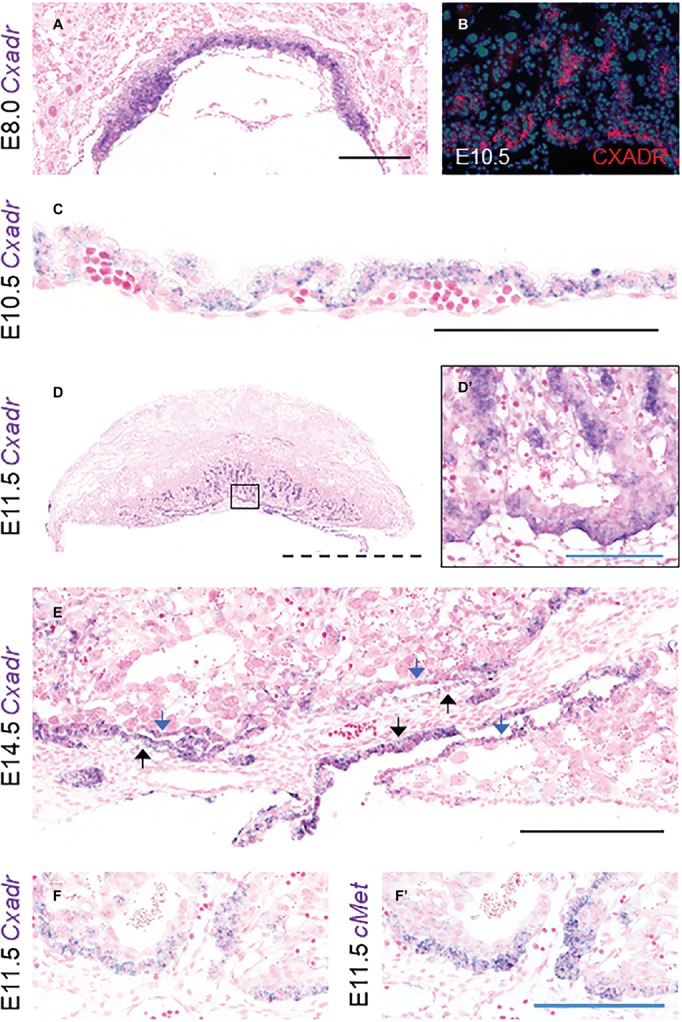
*Cxadr* is expressed in chorion, labyrinth trophoblast, and extraembryonic yolk sac. **(A)** *In situ* hybridization of *Cxadr* in basal chorion cells at E8.0. **(B)** Immunostaining for CXADR in placental labyrinth at E10.5. **(C)** *Cxadr* expression in visceral endoderm cells of the yolk sac at E10.5. **(D)** Expression of *Cxadr* in basal labyrinth cells and in clusters of trophoblast cells adjacent to fetal vessels at E11.5. **(D’)** Higher magnification image of inset in D. **(E)** *Cxadr* expression is evident in cells of the intraplacental yolk sac (IPYS) at E14.5. Parietal (blue arrow) and visceral (black arrow) IPYS layers are indicated. **(F,F’)** *In situ* hybridization of *Cxadr* **(F)** and *cMet* **(F’)** in basal chorion cells at E11.5. Scale bars: 100 μm (solid blue), 200 μm (solid black), and 2 mm (dashed).

To assess the ontogeny of *Cxadr* expression in the mouse placenta, mRNA transcripts were localized from E8.0 to E18.5 using *in situ* hybridization (ISH). At E8.0 *Cxadr* is expressed in trophoblast cells of the chorion, adjacent to the fetal-derived allantois (Figure 1A). At E10.5, CXADR protein localization was detected in the chorionic plate and again in small clusters throughout the labyrinth, and *m*RNA transcripts were identified in the outer epithelial layer of the visceral YS (Figures 1B,C). By E11.5, *Cxadr* positive cells are seen in the chorionic plate and in small clusters throughout the labyrinth, replicating immunofluorescent (IF) findings (Figures 1D,D’). Punctate expression of *Cxadr* is observed in the expanding labyrinth at E14.5; however, chorionic plate expression at this time is reduced, corresponding to the gradual loss of this layer (Figure 1E). Endoderm cells of the intra placental yolk sac (IPYS) are also positive for *Cxadr* transcripts at E14.5, persisting until E18.5 (Figure 1E, E18.5 data not shown). Within the placenta proper, the expression pattern of *Cxadr* is reminiscent to that of the proposed labyrinth progenitor cell marker *cMet* (Figures 1F,F’; Ueno et al., 2013). Expression of *Cxadr* within the developing embryo has previously been reported in numerous organs and tissues, outlined in Supplementary Table S2, but most notably include the developing cardiovascular and lymphatic systems (Dorner et al., 2005; Chen et al., 2006; Mirza et al., 2012). Importantly, CXADR is not perceptibly expressed by fetal endothelial cells (FECs) of the placenta at any time point analyzed and is in line with previously published data (Dorner et al., 2005).

### *CXADR* Is Required for Mid-Gestation Placental Development and Embryonic Survival

To determine whether *Cxadr* null embryos have a placental phenotype, we initially made use of an ENU mutagenesis model containing a C → A mutation at amino acid 210, introducing a premature stop codon (Y → Stop) just prior to the transmembrane domain of the CXADR protein (Supplementary Figure S1). CXADR was efficiently inactivated, shown by reduced *m*RNA and protein expression in mutant placentas (Supplementary Figure S1). Initial dissections of *Cxadr-*ENU embryos and placentas were carried out at E12.5, and in all cases, *Cxadr*^210/210^ homozygous embryos were found deceased, in line with previous reports (Asher et al., 2005; Dorner et al., 2005; Chen et al., 2006; Lim et al., 2008). Dissections carried out 1 day earlier at E11.5 showed that the majority of *Cxadr* null embryos still had an observable heartbeat, and analysis of *Cxadr*^210/210^ placentas was therefore carried out at E10.5 and E11.5, prior to embryonic death (Supplementary Table S8).

**Figure 2:**
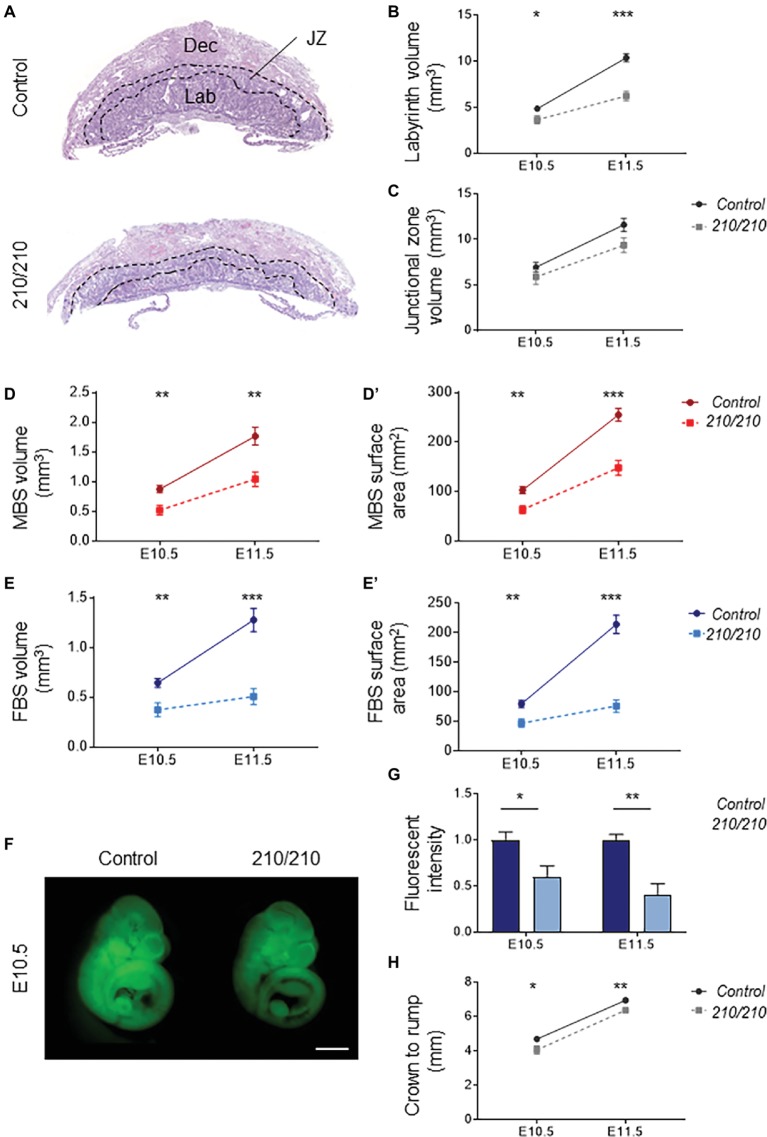
CXADR is required for proper placental development. **(A)** Hematoxylin and eosin stained histological sections of *Cxadr*^+/+^ and *Cxadr*^210/210^ placentas at E11.5. Maternal decidua (Dec), labyrinth (Lab), and junctional zone (JZ) are indicated by dashed outlines. **(B)** Quantification of labyrinth volume. Plots represent the mean ± SEM for a minimum of *n* = 7 placentas per genotype. **(C)** Quantification of junctional zone volume. (**D,D’**) Quantification of maternal blood space volume **(D)** and surface area **(D’)**. **(E)** Quantification of fetal blood space volume **(E)** and surface area **(E’)**. **(F,G)** Whole-mount images of trans-placental passage of rhodamine 123 in embryos at E10.5 **(F)** and quantification **(G)** of fluorescent intensity. Plots represent the mean ± SEM for a minimum of *n* = 6 embryos per genotype. **(H)** Measure of crown to rump length. *p < 0.05, **p < 0.01, ***p < 0.001. Scale bar: 1 mm.

Histological comparisons made between midline cross sections of *Cxadr*^+/+^ and *Cxadr*^210/210^ placentas at E11.5 exposed an overall thinness of the labyrinth in mutant samples (Figure 2A). To define the extent of the labyrinth deficit, Cavalieri’s principle was used to determine placental morphometry at E10.5 and E11.5, revealing *Cxadr*^210/210^ labyrinth volumes were markedly reduced at E10.5, increasing in severity by E11.5 (Figure 2B; Coan, 2004). It is noted that following E10.5 there is no substantial growth or expansion achieved in this region. Alternatively, junctional zone (JZ) volumes showed no significant differences at either time point (Figure 2C), confirming deficits were restricted to the labyrinth compartment. Stereology was also performed on heterozygous placentas, and no differences between *Cxadr*^+/+^ and *Cxadr*^+/210^ were noted for any measured parameters; figures and graphs therefore depict *Cxadr*^+/+^ and *Cxadr*^210/210^ examples for simplicity.

About two thirds of gene knockouts with placental phenotypes describe labyrinth defects. By far the most common labyrinth defect involves under-development of the region, revealing decreased fetal vessel branching or thickened IHM layers, with severe intrauterine growth restriction (IUGR) or fetal loss due to absent or insufficient metabolic exchange (Watson, 2005; Cross et al., 2006). Significantly reduced volumes are seen for maternal and fetal blood spaces within the *Cxadr* mutant labyrinth, beginning at E10.5 (Figures 2D,E). Similarly, decreased surface areas were observed in both maternal and fetal vessels, illustrating a considerably diminished IHM area available for exchange (Figures 2D’,E’). To this end, trans-placental passage was assessed by injection of the fluorescent dye rhodamine 123 into the maternal circulation and measurement of dye accumulation within the embryo (Dupressoir et al., 2009); results are consistent with stereological findings, revealing markedly decreased IHM transfer of rhodamine 123 occurring as early as E10.5 and progressively worsening by E11.5 (Figures 2F,G). A mild reduction in crown-rump lengths was observed in *Cxadr* mutant embryos at both E10.5 and E11.5 (Figure 2H).

**Figure 3:**
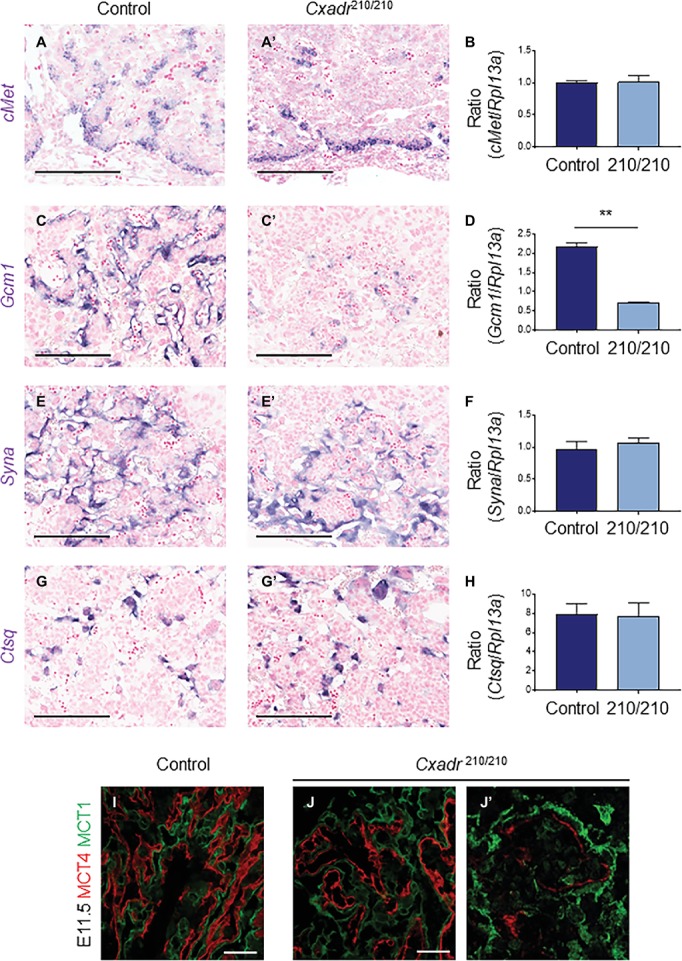
CXADR is required for appropriate expansion of labyrinth trophoblast cell types. *In situ* hybridization in *Cxadr*^+/+^ and *Cxadr*^210/210^ placentas at E11.5 for *cMet* **(A,A’)**, *Gcm1* **(C,C’)**, *Syna* **(E,E’)**, and *Ctsq* **(G,G’)**. qRT-PCR analysis for *cMet* **(B)**, *Gcm1* **(D)**, *Syna* **(F)**, and *Ctsq* **(H)** relative to *Rpl13a*. Plots represent the mean ± SEM for a minimum of *n* = 7 placentas per genotype. **(I,J)** Immunofluorescence of MCT1 (green) marking syncytial layer I cells, and MCT4 (red) marking syncytial layer II cells in *Cxadr*^+/+^ **(I)** and *Cxadr*^210/210^ **(J)** labyrinth vessels at E11.5. **(J’)** Higher magnification image of *Cxadr*^210/210^ peripheral labyrinth vessel loss. **p < 0.01. Scale bars: 200 μm (black) and 50 μm (white).

To assess the trophoblast component of the labyrinth, spatial distribution of cell subtypes was assessed by marker analysis at E11.5 and carried out by ISH on midline cross sections*. cMet* positive cell clusters, marking putative labyrinth progenitors, and the same cells expressing *Cxadr*, including basal chorion cells and clusters of cells adjacent to branching points in the labyrinth, are less evident throughout the *Cxadr*^210/210^ labyrinth. Interestingly, *m*RNA transcript levels detected by qRT-PCR within whole placental samples are not significantly different, suggesting a possible compensatory upregulation in *cMet* expression in the remaining positive cells of the chorion (Figures 3A,B). *Gcm1* expression, marking branching points and nascent SynT-II cells, is reduced at this time point, evident by both ISH and qRT-PCR (Figures 3C,D), suggesting CXADR is required following E10.5 for the proper expansion and development of SynT-II. However, important growth factors known to be associated with maintaining *Gcm1* and syncytial layer II development, including hepatocyte growth factor (*Hgf*) and various *Wnt* proteins, showed no change in expression between genotypes (Supplementary Figure S2; Matsuura et al., 2011; Lu et al., 2013; Ueno et al., 2013; Zhu et al., 2017). In addition, markers of SynT-I (*Syna*) and sinusoidal-trophoblast giant cells (S-TGCs; *Ctsq*) do not show reduced or altered expression (Figures 3E–H).

MCT1 (encoded by *Slc16a1*) and MCT4 (encoded by *Slc16a3*) are monocarboxylate (lactate) transporters found on the apical plasma membrane (maternal side) of SynT-I and basal membrane (fetal side) of SynT-II, respectively (Nagai et al., 2010). In addition to marking the individual syncytial layers, these proteins also play an important transport role in the mouse placenta, and their expression is suggested to be a useful measure of IHM transport capacity (Moreau et al., 2014). While MCT1 and MCT4 protein localization in *Cxadr*^+/+^ E11.5 placental sections show the normal distribution of interdigitated SynT-I and SynT-II cells, *Cxadr*^210/210^ labyrinths display altered IHM organization (Figures 3I,J), with peripheral areas (Figure 3J’) showing fetal capillary damage and a loss of SynT-II marker MCT4. Adjacent to decreased MCT4 expression, the intensity of MCT1 expression in SynT-I remains intact (Figure 3J). These results corroborate ISH findings, although *Gcm1* loss at E11.5 was more widespread, possibly reflecting an earlier cessation to branching morphogenesis reflecting its role as a regulator of SynT-II differentiation (Figures 3C,D).

### *Cxadr* Null Mice Display Impaired Vascular Expansion and Defective IHM Morphogenesis During Labyrinth Development

**Figure 4:**
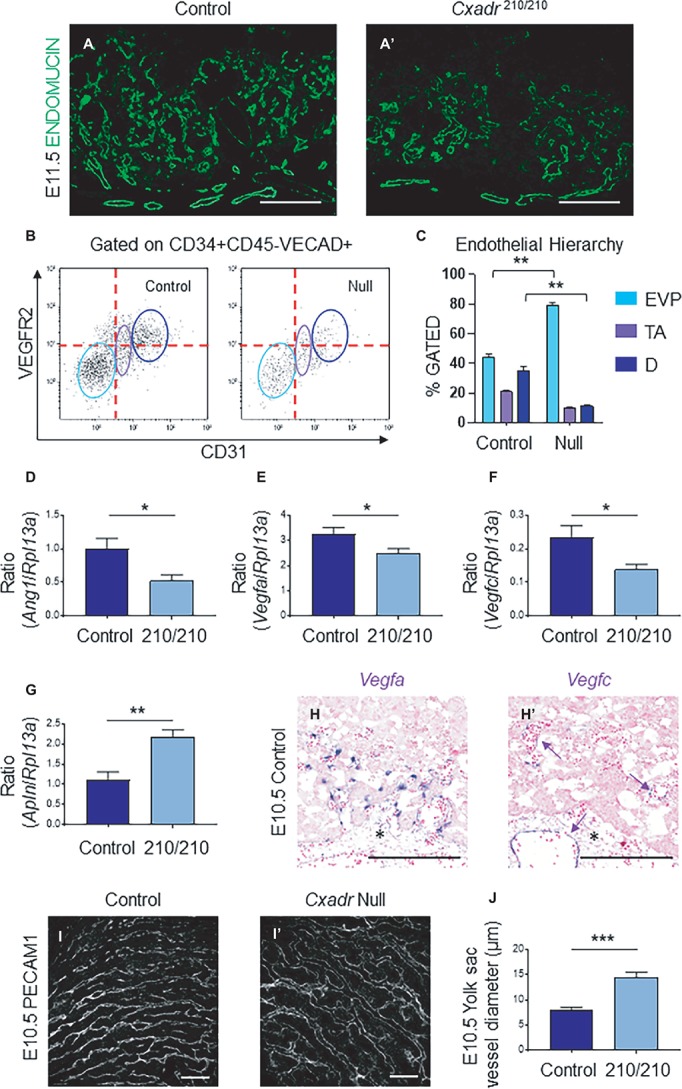
*Cxadr* null mice display impaired vascular invasion and defective IHM morphogenesis. **(A)** Immunofluorescence of ENDOMUCIN (green) marking fetal endothelial cells in *Cxadr*^+/+^ **(A)** and *Cxadr*^210/210^ **(A’)** labyrinth sections at E11.5. **(B)** CD34+ CD45− VECAD+ gated labyrinth cells reveal distinct endothelial populations in *Cxadr*^+/+^ and *Cxadr*^Δ/Δ^ samples, based on VEGFR2 and CD31 expression. From left to right: endovascular progenitors (EVP), transit amplifying (TA), FIGURE 4and differentiated (D) endothelial cells. Minimum of *n* = 2 labyrinths per genotype were pooled per litter, and comparisons were made between *n* = 3 litters. **(C)** Endothelial hierarchy within each gated population/genotype. **(D–F)** qRT-PCR analysis for *Ang1*, *Vegfa,* and *Vegfc* expression in E11.5 placentas relative to *Rpl13a*. Plots represent the mean ± SEM for a minimum of *n* = 7 placentas per genotype. **(G)** qRT-PCR analysis for *Apln* expression in E11.5 placentas relative to *Rpl13a*. Plots represent the mean ± SEM for a minimum of *n* = 7 placentas per genotype. **(H)** *In situ* hybridization of *Vegfa* in labyrinth cells adjacent to fetal vessels at E10.5. **(H’)** *In situ* hybridization of *Vegfc* in some (arrow), not all, fetal endothelial cells of the labyrinth and allantois at E10.5. **(I)** Immunofluorescence of PECAM1 marking endothelial cells in *Cxadr*^+/+^ **(I)** and *Cxadr*^Δ/Δ^ **(I’)** flat mount yolk sacs at E10.5. **(J)** Quantification of yolk sac vessel diameter at E10.5. Plots represent the mean ± SEM for *n* = 5 yolk sacs per genotype. *p < 0.05, **p < 0.01, ***p < 0.001. Scale bars: 200 μm (white) and 300 μm (black).

In the labyrinth of *Cxadr* null placentas, the initial formation of fetal blood vessels occurs unhindered. However, by E10.5 decreased fetal blood space volume and surface area are observed, suggesting drivers of IHM development are perturbed soon after the placental, embryonic, and yolk sac vasculatures interconnect (Figures 2E,E’). Recently, endothelial cells of the mouse placenta were found to be a heterogeneous population that can be grouped into endovascular progenitors (EVP), transit amplifying (TA), and terminally differentiated (D) cell types based on their levels of expression of CD34, VECAD, CD31, VEGFR2, and their lack of hematopoietic marker CD45 (Patel et al., 2017). This suggests neovessel formation in the placenta involves the sequential differentiation of EVP cells to form a definitive vascular network comprised of TA and mature D endothelial cells (Patel et al., 2017). The extent of diminished vascularization in *Cxadr*^210/210^ labyrinths is easily observed at E11.5 by IF detection of ENDOMUCIN (marking placental endothelial cells; Figures 4A,A’). In line with this, isolated endothelial cells from E11.25 dissociated labyrinths reveal a reduction in total endothelial cell numbers in *Cxadr* null samples (Figure 4B). Interestingly, within this population, EVP cells denote the majority of endothelial cells present in *Cxadr* null labyrinths, with very little TA and terminally differentiated endothelium represented (Figure 4C). This suggests a reduced functional capacity of EVP to differentiate into mature endothelial cells, which form the basis of the vascular network.

To elucidate the basis for poor growth of the placental vasculature, analyses of gene expression of angiogenic signaling factors were assessed. Results revealed significant decreases in pro-angiogenic angiopoietin 1 (*Ang1*), vascular endothelial growth factor a (*Vegfa*) and *Vegfc*, in conjunction with increased apelin (*Apln*) in placental samples at E11.5 (Figures 4D–G). While no changes were observed for placental growth factor (*Pgf*), neuropilin 1 (*Nrp1*), *Apela*, *Ang2*, *Vegfb*, *Vegfd,* or change in the expression of hypoxia inducible factor-1α (HIF-1α) and EGF-like domain containing protein 7 (*Egfl7*), as part of a hypoxic response (Supplementary Figure S2; Lacko et al., 2014; Ho et al., 2017). Localization of *Vegfa* and *Vegfc* mRNA in wild-type labyrinths revealed *Vegfa* expression in basal chorion cells at E9.5, and an unknown cell type closely associated with labyrinth fetal vessels at E9.5 and 10.5, potentially a support or mural cell (Supplementary Figure S3, Figure 4H). By contrast, *Vegfc* is expressed in a small subset of fetal endothelial vessels, possibly suggesting an association with venule versus arteriole subtypes (Supplementary Figure S3, Figure 4H’). Reciprocally, while expression of the APJ receptor (*Aplnr*) is observed in the majority of labyrinth vasculature, it is notably absent in several vessels (Supplementary Figure S3). Interestingly, while there was a decrease in FECs, there was no accompanying change in expression for any associated angiogenic tyrosine-kinase (*Vegfr1*, *Vegfr2*, *Tie1*, *Tie2*) or G-protein coupled receptors (*Aplnr*) in *Cxadr* null placentas compared to wild type (Supplementary Figure S2).

The cell-death marker, active (cleaved) Caspase-3 (CASP3), was used to determine whether apoptosis was occurring in IHM cells. At E10.5 and E11.5 immunofluorescence reveals an increase in apoptotic cells in the labyrinth of *Cxadr*^210/210^ placentas compared to controls (Supplementary Figure S4), including a number of CASP3 positive cells seen throughout the allantois (data not shown). In addition, double IFs comparing expression of ENDOMUCIN (marking FECs) and CASP3 at E11.5 indicate some of the cells undergoing active cell death include endothelial cells (Supplementary Figure S4). In line with previous results, SynT-I cells remain unaffected, with no apoptosis or cell loss observed in MCT1-positive cells (Supplementary Figure S4).

Due to the emerging vascular phenotype, the impact of the *Cxadr* mutation was further investigated in the yolk sac (YS) vasculature. Examination of E10.5 YS vessels by whole-mount immunofluorescence for the endothelial marker platelet endothelial cell adhesion molecule 1 (PECAM 1) revealed wider vessels in *Cxadr* null compared with wild-type YS’s (Figures 4I,I’,J). The larger vessels seen in *Cxadr* null YS suggest that reduced vascular remodeling is occurring, complementing the growth restriction observed in the embryo at this time (Lucitti et al., 2007; Udan et al., 2013; Garcia and Larina, 2014).

### Placental Defects in *Cxadr* Null Mice Are Secondary to Embryonic Development

Due to the broad nature of *Cxadr* expression (placental, YS and embryonic), it cannot be determined with a global loss of *Cxadr* within which tissue compartment CXADR is critical. However, Tg^*Sox2*-Cre^ mice have been widely used to conditionally knockout genes within the embryo while retaining unaltered gene expression within trophoblast cell types (Hayashi et al., 2003). We obtained *Cxadr*^f/f^ mice to facilitate conditional gene deletion (Pazirandeh et al., 2011), but first generated *Cxadr*^+/Δ^ mice and crossed them to ensure the phenotypes of *Cxadr*^Δ/Δ^ placentas were consistent with what we observed in *Cxadr*^210/210^ placentas (Supplementary Figure S5). We found *Cxadr*^Δ/Δ^ placentas phenocopy *Cxadr-*ENU mutants, indicating the ENU mutation and the knockout allele are functionally equivalent (Supplementary Figure S6).

We also utilized the Cre reporter mouse line B6.Cg-*Gt(ROSA)26Sor*^tm9(CAG-tdTomato)Hze^/J (herein referred to as “tdTom”; Madisen et al., 2009) to confirm the reported patterns of Tg^*Sox2*-Cre^ activity in our hands. TdTom expression was observed in the visceral yolk sac (mosaic in the endoderm layer), allantois, and labyrinth FECs at E14.5, with Cre activity notably absent from the trophoblast compartment where *Cxadr* is expressed (Supplementary Figure S7). Within the embryo proper, the vast majority of cells at E14.5 are positive for tdTom expression, reflecting early epiblast expression of Cre from the *Sox2* promoter (data not shown).

**Figure 5:**
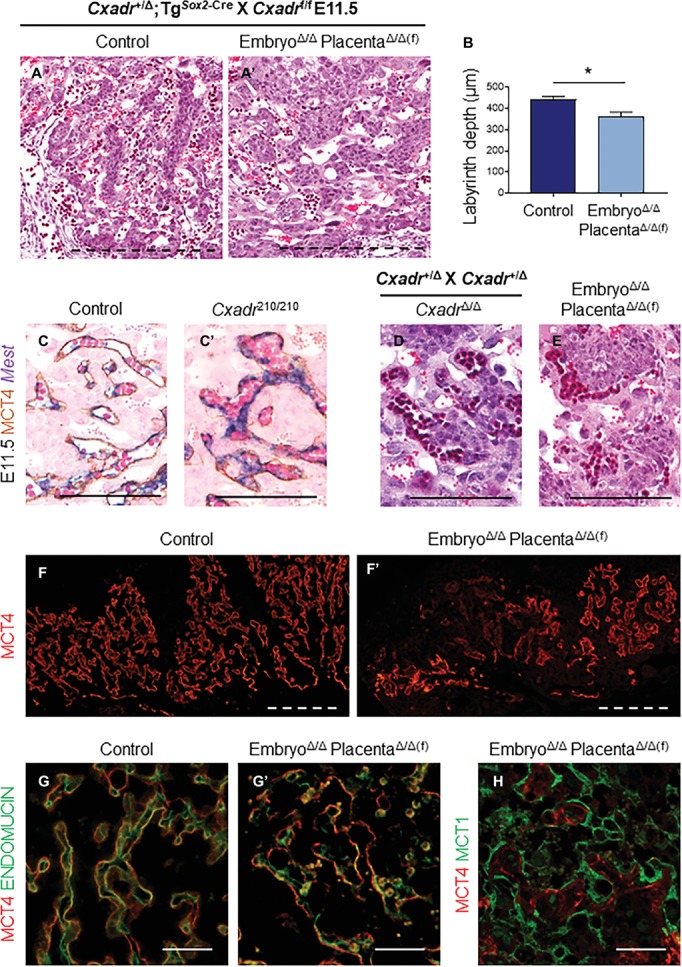
Placental defects in *Cxadr* null mice are secondary to embryo development. **(A)** Hematoxylin and eosin stained histological sections of control (*Cxadr*^+/Δ^;Tg^*Sox2*-Cre^) (A) and *Cxadr*^Δ/Δ(f)^;Tg^*Sox2*-Cre^ **(A’)** labyrinths at E11.5. **(B)** Average labyrinth depth. Plots represent the mean ± SEM for *n* = 4 placentas per genotype. **(C)** *In situ* hybridization for *Mest* and immunohistochemistry for MCT4 in *Cxadr*^+/+^ (C) and *Cxadr*^210/210^ **(C’)** peripheral interhaemal membrane structures at E11.5. **(D,E)** Hematoxylin and eosin stained histological sections of *Cxadr*^Δ/Δ^ (D) and *Cxadr*^Δ/Δ(f)^;Tg^*Sox2*-Cre^ (E) peripheral labyrinth fetal vessels at E11.5. **(F)** Immunofluorescence of MCT4, marking syncytial layer II, in control (*Cxadr*^+/Δ^;Tg^*Sox2*-Cre^) (F) and mutant (*Cxadr*^Δ/Δ(f)^;Tg^*Sox2*-Cre^) **(F’)** E11.5 placentas. **(G)** High magnification images showing immunofluorescence of MCT4 (red) and ENDOMUCIN (green), marking fetal endothelial cells, in control (*Cxadr*^+/Δ^;Tg^*Sox2*-Cre^) (G) and mutant (*Cxadr*^Δ/Δ(f)^;Tg^*Sox2*-Cre^) **(G’)** labyrinth sections at E11.5. **(H)** Immunofluorescent detection of MCT4 (red) and MCT1 (green), marking syncytial layer I, in E11.5 *Cxadr*^Δ/Δ(f)^;Tg^*Sox2*-Cre^ labyrinth sections. *p < 0.05. Scale bars: 300 μm (dashed black), 100 μm (solid black), 200 μm (dashed white), and 50 μm (solid white).

We then bred *Cxadr*^+/Δ^;Tg^*Sox2*-Cre^ males with *Cxadr*^f/f^ females to create *Cxadr*^Δ/Δ^;Tg^*Sox2*-Cre^ embryos with placentas that retain a floxed *Cxadr* allele (*Cxadr*^Δ/Δ(f)^;Tg^*Sox2*-Cre^) due to an absence of trophoblast *Sox2*-Cre activity. If placental defects are the primary reason for *Cxadr*^Δ/Δ^ embryonic phenotypes, we would expect to see ameliorated cardiovascular development in *Cxadr*^Δ/Δ^ embryos and survival past E13.5. However, counter to our original hypothesis, *Cxadr*^Δ/Δ^;Tg^*Sox2*-Cre^ embryos connected to heterozygous placentae died at the same gestational age as *Cxadr-*ENU and global knockout lines (between E11.5-E12.5). Despite sparing placental trophoblast from Cre-mediated deletion of *Cxadr*, histological analysis of *Cxadr*^Δ/Δ(f)^;Tg^*Sox2*-Cre^ placentas surprisingly showed altered IHM architecture and reduced IHM branching reminiscent of global knockout placentas (Figures 5A,A’). Overall, heterozygous placentas attached to *Cxadr* null embryos appear flatter and have a significantly reduced labyrinth depth, as in *Cxadr*^210/210^ and *Cxadr*^Δ/Δ^ samples (Figure 5B). Higher magnification images show comparable congested peripheral vessels, where blood flow appears to be disrupted, in *Cxadr*^210/210^, *Cxadr*^Δ/Δ^, and *Cxadr*^Δ/Δ(f)^;Tg^*Sox2*-Cre^ labyrinth sections (Figures 5C–E). Furthermore, disorganized IHM branching coupled with areas of SynT-II loss is illustrated by IF localization of MCT4 (Figures 5F,F’). Disrupted MCT4 expression is also coupled with the loss of FEC marker ENDOMUCIN while retaining SynT-I marker MCT1 (Figures 5G–H). These results indicate that labyrinth damage is secondary to an embryonic or yolk sac requirement for *Cxadr*.

### Heart-Specific Deletion of *Cxadr* Does Not Impact Placental Development

**Figure 6:**
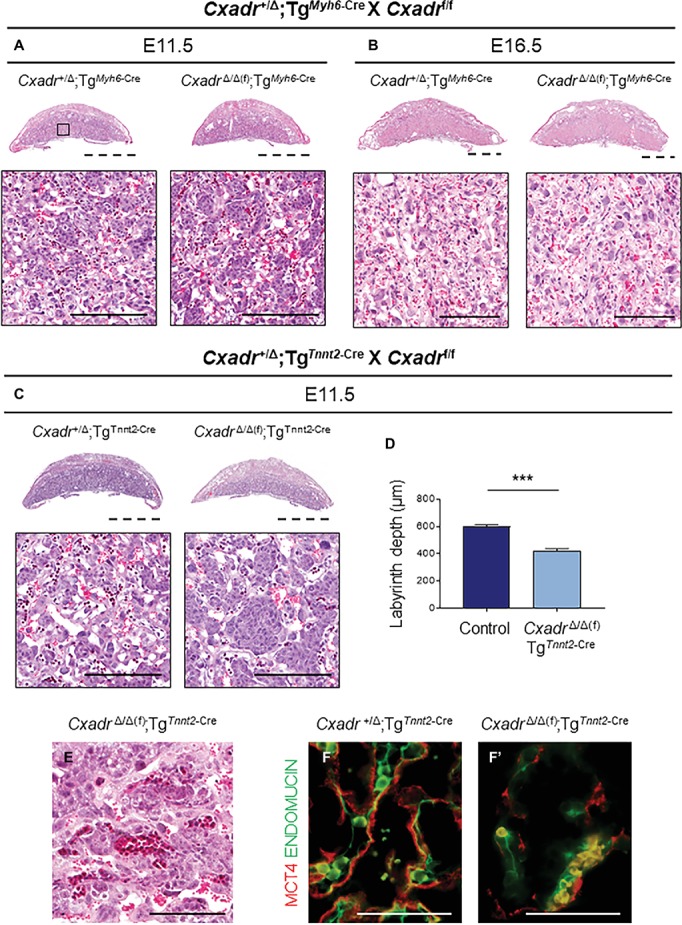
Heart specific deletion of *Cxadr* does not impact placental development. **(A,B)** Hematoxylin and eosin stained histological sections of control (*Cxadr*^+/Δ^;Tg^*Myh6*-Cre^) and mutant (*Cxadr*^Δ/Δ(f)^;Tg^*Myh6*-Cre^) placentas at E11.5 **(A)** and E16.5 **(B)**. Higher magnification of inset depicts close up of labyrinth. **(C)** Hematoxylin and eosin stained histological sections of control (*Cxadr*^+/Δ^;Tg^*Tnnt2*-Cre^) and mutant (*Cxadr*^Δ/Δ(f)^;Tg^*Tnnt2*-Cre^) placentas at E11.5. **(D)** E11.5 control (*Cxadr*^+/Δ^;Tg^*Tnnt2*-Cre^) and mutant (*Cxadr*^Δ/Δ(f)^;Tg^*Tnnt2*-Cre^) labyrinth depths. Plots represent the mean ± SEM for *n* = 4 placentas per genotype. **(E)** Hematoxylin and eosin stained histological section of *Cxadr*^Δ/Δ^(f); Tg^*Sox2*-Cre^ peripheral labyrinth fetal vessels at E11.5. **(F)** High magnification images showing immunofluorescence of MCT4 (red), marking syncytial layer II, and ENDOMUCIN (green), marking fetal endothelial cells, in control (*Cxadr*^+/Δ^;Tg^*Tnnt2*-Cre^) **(F)** and mutant (*Cxadr*^Δ/Δ(f)^;Tg^*Tnnt2*-Cre^) **(F’)** labyrinth sections at E11.5. ***p < 0.001. Scale bars: 2 mm (dashed black), 100 μm (solid black), and 50 μm (solid white).

Since a loss of trophoblast *Cxadr* is not responsible for the placental phenotypes observed in *Cxadr* null mutants, primary heart defects may underlie the observed impact on placentation. Therefore, to query the placenta-heart axis in this inverse direction, heart-specific deletion of *Cxadr* (driven by *Myh6*-Cre and *Tnnt2*-Cre transgenes) was employed. Tg^*Myh6*-Cre^ driven deletion of *Cxadr* is not lethal and pups born from such crosses survive to adulthood. In line with previous reports, the majority of *Cxadr*^Δ/Δ(f)^;Tg^*Myh6*-Cre^ embryos were detected alive in litters collected at E11.5, E13.5, and E16.5, and histological analysis found no obvious labyrinth defects in these animals at corresponding stages (Figures 6A,B; Supplementary Table S9; Chen et al., 2006; Lim et al., 2008; Kallewaard et al., 2009). Alternatively, crosses of *Cxadr*^+/Δ^;Tg^*Tnnt2*-Cre^ and *Cxadr*^f/f^ mice resulted in embryonic lethality by E12.5, coupled with similar placental phenotypes to the Tg^*Sox2*-Cre^ conditional and global knockout models (Figure 6C–F). Histological and IF analysis at E11.5 again revealed thinner placentas with decreased labyrinth depth, altered fetal branching, altered peripheral vessels, and a loss of fetal endothelium coupled with a breakdown of SynT-II in placentas of *Cxadr*^Δ/Δ(f)^;Tg^*Tnnt2*-Cre^ embryos (Figures 6C–F’). On closer inspection, FECs do not appear adjacent or in contact with SynT-II as both layers are lost (Figure 6F’).

Interestingly, when we examined *Myh6*-Cre and *Tnnt2*-Cre activity in the placenta, using the tdTom Cre reporter line, we similarly found diverse results. While Tg^*Myh6*-Cre^ driven expression of tdTom was not detected in E16.5 placentas (data not shown), whole-mount images and sections from Tg^*Tnnt2*-Cre^;Tg^tdTom^ placentas depict tdTom positive cells in the labyrinth, with a staining pattern reminiscent of S-TGCs, and in an unknown cell type(s) within the JZ, likely to be Channel-TGCs (Supplementary Figure S8; Rai and Cross, 2014). Double IFs for ENDOMUCIN, marking FECs, and MCT4, marking SynT-II, do not reveal any overlapping expression between tdTom positive cells and these layers in the labyrinth (Supplementary Figure S8). Cells positive for tdTom are also seen around the umbilical vessels and throughout the fetal-derived allantoic mesenchyme and YS (Supplementary Figure S8). Moreover, additional tissues containing *Tnnt2*-Cre activity were observed throughout the embryo proper. Whole-mount images and sections through the embryo reveal *Tnnt2*-Cre positive cells at E16.5 are found in the liver, lips, skeletal muscle, tongue, bladder, and throughout the CNS (Supplementary Figure S9, section data not shown). *Tnnt2*-Cre induced deletion of *Cxadr* results in both embryonic lethality and importantly placental defects. However, the expression pattern of this transgene within the placenta does not align with *Cxadr* expression and therefore implicates areas of *Tnnt2* and *Cxadr* overlap within the embryo or YS to be responsible for the secondary placental phenotypes observed.

## Discussion

CXADR expression has been cursorily reported in trophoblast (Dorner et al., 2005), but the precise cellular localization and function within this tissue are unknown. Since impairments in placental development and function can cause secondary cardiovascular abnormalities, a phenomenon referred to as the placenta-heart axis, we hypothesized that placental phenotypes may underlie or contribute to the reported cardiovascular defects and embryonic lethality observed in *Cxadr* mutant embryos.

In the current study, we find that homozygous deletion of *Cxadr*, a mutation known to cause developmental heart and lymphatic defects, also causes severe defects in placental morphogenesis prior to embryonic lethality. Specifically, inactivation of *Cxadr* results in insufficient expansion of endothelial populations within the labyrinth. While it is clear an initial fetal vessel network forms, overall elaboration and patterning of a functional IHM in *Cxadr* mutants is significantly hampered, decreasing capacity for adequate placental transport. Due to the timing and severity of these placental phenotypes, placental insufficiency may play a key role in the demise of *Cxadr* mutants and may in part explain the variability observed in the reported embryonic heart defects by different studies (Asher et al., 2005; Dorner et al., 2005; Chen et al., 2006; Lim et al., 2008; Kallewaard et al., 2009).

### FEC-SynT-II Interactions Are Essential for Proper Morphogenesis of the IHM

Observations from numerous KO mouse models have demonstrated that reciprocal molecular crosstalk between trophoblast and allantoic-derived cells is essential for normal labyrinth morphogenesis and expansion (reviewed in (Hemberger and Cross, 2001; Rossant and Cross, 2001; Watson, 2005; Cross et al., 2006)). One interesting observation from *Cxadr* mutant placentas is the synchronized loss of FECs and SynT-II cells. The sensitivity of SynT-II to the biochemical and molecular cues, conveyed by neighboring endothelium, has a profound influence on placental morphogenesis. For example, WNTs emanating from allantoic cells act through the FZD5 receptor on SynT-II cells to maintain *Gcm1* expression, a transcription factor essential for SynT-II differentiation and IHM expansion (Lu et al., 2013). In *Cxadr* mutant placentas, *Gcm1* expression is not maintained, and FEC differentiation is severely affected, indicating a breakdown in this important FEC-SynT-II dialogue. Intriguingly, the two syncytiotrophoblast layers in the labyrinth are joined by gap junctions and are thought to operate as a single unit providing an apical (SynT-I) and basal (SynT-II) layer for active and facilitated diffusion of nutrients (Coan et al., 2005; Nagai et al., 2010); these layers are quite often considered one entity functionally. It is surprising then that the loss of SynT-II does not result in the loss of SynT-I or S-TGCs. The relationship between SynT-II and FECs appears significantly more interdependent, and it would seem that the affiliation SynT-II and FECs have with the molecules and signals being relayed to each other, or from neighboring support cells, bind trophoblast and fetal-derived components of the IHM together, directing branching morphogenesis of the placental fetal vasculature.

Recently, a hierarchy among endothelial cells has been described in adult tissues and in the placenta of mice, including EVP, TA, and definitively differentiated (D) endothelial cells, with EVP cells providing a potential pool of tissue resident vascular stem cells (Patel et al., 2017). Of note, the majority of endothelial cells within the vascular population of *Cxadr* mutant placentas are EVPs, raising the question of whether a block in labyrinth endothelial differentiation/development is the catalyst for poor IHM expansion. Importantly, expression of *Wnts* and other growth factors known to be critical for labyrinth formation and SynT-II maintenance are not altered in *Cxadr* mutant placentas (Supplementary Figure S2), implicating a breakdown in pathways not previously linked with FEC-SynT-II communication. However, decreases in the expression of several additional angiogenic factors (*Vgefa, Vegfc*, *Ang1,* and *Apln*), some of which are required for the growth and maintenance of placental endothelial cells as well as trophoblast behaviors in both mouse and human placentas (Ahmed and Perkins, 2000; Dunk et al., 2000; Burton et al., 2009; Chen and Zheng, 2014; Kappou et al., 2015), were observed in *Cxadr* mutant labyrinths.

The precise roles these factors play in IHM development are currently unknown; however, the different expression patterns of *Vegfa* and *Vegfc* suggest diverse functions for these angiogenic factors within the developing placenta (Supplementary Figure S3). In the labyrinth, *Vegfa* is expressed in chorion trophoblast and in an unknown cell type adjacent to FECS (likely mural cells) in a prime position to instruct endothelial cell expansion, while expression of *Vegfc* appears limited to a subset of fetal endothelial vessels (Supplementary Figure S3). Interestingly, disruption of key angiogenic pathways, including VEGFA and C, has causal links to IUGR and abnormal villous development in mouse and human pregnancies (Ahmed and Perkins, 2000; Dunk et al., 2000; Burton et al., 2009; Chen and Zheng, 2014; Kappou et al., 2015). Additionally, the *Apln* ligand and its receptor are reportedly expressed in all endothelial arteries, and *Apln* is also noted to have widespread expression in extraembryonic tissues, including endothelial tip cells of the developing labyrinth (Ho et al., 2017; Mughal and O’Rourke, 2018). During development, *Apln* is important for angiogenesis, and the role of the apelinergic system during this process can be either detrimental or beneficial, depending on the underlying pathology (He et al., 2015; Mughal and O’Rourke, 2018). It is unclear whether the increase in *Apln* expression noted here may be playing a damaging or compensatory role.

### Extra-Placental *Cxadr* Expression Is Critical for Mid-Gestation Placental Labyrinth Morphogenesis

Placental abnormalities and embryonic lethality frequently co-exist (Perez-Garcia et al., 2018), and increasing evidence from animal models shows that primary placental defects in particular can influence cardiogenesis (aka the placenta-heart axis), although the basis of the mechanistic relationship between these two concurrently developing organs has not been fully revealed (Hemberger and Cross, 2001; Linask, 2013; Burton and Jauniaux, 2018). Due to the nature of their physical connection, differentiating between the possible causes of damage such as nutrient and endocrine deficiency, co-factor availability or local hemodynamic impacts prove difficult. Furthermore, the characterization of secondary effects on the placenta in cases of primary cardiovascular phenotypes has not been previously elucidated.

Despite *Cxadr* expression in labyrinth trophoblast progenitors, and the severe placental abnormalities that develop in the absence of *Cxadr*, retention of trophoblast *Cxadr* expression with deletion of *Cxadr* outside the placental trophoblast compartment, using *Sox2-Cre* mice (Hayashi et al., 2003), confirmed that the observed placental phenotypes in *Cxadr* mutants are a consequence of primary embryonic or non-trophoblast extraembryonic defects. While it is true, the functional capacity of the placenta can impose mechanical constraints that affect blood flow patterns (Linask et al., 2014; Burton et al., 2016); the heart is the primary contributing factor to hemodynamics (pressure, shear stress, and cyclic strain), producing and organizing local and global molecular and cellular changes within the whole vascular system, not just the embryo proper. Previous studies reported *Cxadr* mutants die due to cardiac defects. Therefore, the persistence of a placental phenotype in heterozygous placentas attached to *Cxadr* null embryos suggested that altered cardiac output or failing heart function could be the origin of these secondary placental defects. In an attempt to address the impact of altered cardiac development on the placenta and assess the two-way nature of the placenta-heart axis, we sought to conditionally delete CXADR during gestation. However, when *Cxadr* is deleted using Tg^*Myh6*-Cre^, the most heart-restricted gene utilized in these studies, embryos can survive to adulthood, and we observed no significant placental phenotypes (Chen et al., 2006; Lim et al., 2008; Kallewaard et al., 2009). Therefore, any cardiac phenotypes caused by *Myh6*-Cre induced deletion of *Cxadr* are not critical during development or sufficient enough to cause obvious secondary placental defects, probably accounting for embryo survivability.

Contrary to this, deletion of *Cxadr* using *Tnnt2*-Cre mice is not compatible with embryonic survival (Chen et al., 2006). Accordingly, we found placental phenotypes following Tg^*Tnnt2*-Cre^ driven deletion of *Cxadr* that resemble those of the more broadly expressed Tg^*Sox2*-Cre^ and global deletion models. The difference in embryonic phenotypes between Tg^*Myh6*-Cre^ and Tg^*Tnnt2*-Cre^ driven deletion of *Cxadr* was originally hypothesized to be the result of a later onset of Cre expression in the hearts of *Myh6*-Cre mice compared with *Tnnt2*-Cre mice (Chen et al., 2006), reflecting a critical developmental window for CXADR function in the heart prior to E11.5. However, we observed Cre activity in the hearts of *Myh6*-Cre mice as early as E9.0 with *m*RNA expression detected at E8.5 by ISH, refuting this notion (Supplementary Figure S10). If loss of *Cxadr* in the heart is the driver of secondary placental defects, it is due to the wider expression of *Tnnt2*-Cre throughout the myocardium compared with *Myh6*-Cre (Yan et al., 2016), rather than differences in the developmental onset of Cre expression. This raises an interesting question, could the degree of cardiac dysfunction, due to variation in the percentage of cardiomyocytes that express *Cxadr*, result in the difference between embryonic lethality, as seen in *Cxadr*^Δ/Δ(f)^;Tg^*Tnnt2*-Cre^ embryos, and cardiomyopathies like those exhibited in *Cxadr*^Δ/Δ(f)^;Tg^*Myh6*-Cre^ adult mice? (Lim et al., 2008). However, confounding our analysis, expression of Cre from the Tg^*Tnnt2*-Cre^ transgene is not restricted to the heart and is in fact broader than previously appreciated. Cre expression from the *Tnnt2* promoter is noted in lateral plate mesoderm (LPM) at E7.5 (Wang et al., 2000, 2001), and we observed cells positive for tdTom throughout LPM derivatives, including components of the circulatory system and in the extraembryonic YS (Supplementary Figures S8, S9). Therefore, the cells responsible for the observed placental phenotypes, in the absence of *Cxadr*, are within the non-overlapping cell populations expressing Cre between the Tg^*Myh6*-Cre^ and Tg^*Tnnt2*-Cre^ transgenes, and this may include cardiac, vascular (embryonic or extraembryonic), or vascular support cells.

Within the embryo, dilated aorta and cardinal veins (CVs) have been detected in several *Cxadr* KO studies by E11.5 (Dorner et al., 2005; Chen et al., 2006; Marsman et al., 2014), and Mirza et al. observed deformed and dilated lymphatic vessels resulting from impaired formation of cell-cell junctions in lymphatic endothelium (Mirza et al., 2012). Expression of *Cxadr* in embryonic endothelial cells is somewhat inconclusive but has been reported in several studies (Carson et al., 1999; Vincent et al., 2004), and *Cxadr* expression in lymphatic endothelial cells (LECs) has been shown, with the protein localized to cell-cell junctions (Mirza et al., 2012). LECs bud from the CV, beginning at E10.5, signaling the emergence of the developing lymphatic system. In a wild-type situation, disruption to the CV endothelial barrier is avoided as budding LECs increase expression of cell adhesion markers and remain attached to CV cells *via* cell-cell junctions (Yang and Oliver, 2014). By E11.5 lymph sacs are noted along the posterior of the CV, a loss of proper cell-cell contact in lymph sacs could facilitate a loss of CV integrity and tone. This suggests that a loss of *Cxadr* within cells of the developing embryonic cardiovascular system of *Cxadr*^210/210^, *Cxadr*^Δ/Δ^, *Cxadr*^Δ/Δ(f)^;Tg^*Sox2*-Cre^, *Cxadr*^Δ/Δ(f)^;Tg^*Tnnt2*-Cre^ embryos likely causes primary cardiovascular defects that result in secondary placental phenotypes. However, since Tg^*Sox2*-Cre^ and Tg^*Tnnt2*-Cre^ can drive gene deletions within the YS, we cannot rule out a primary YS defect as a driving or contributing cause of placental defects. Indeed, we observed a loss of vessel tone in *Cxadr* null YSs at E10.5. This is of importance, as dilated vessels are known to correlate with altered blood flow, and perturbed YS development has previously been noted to affect hemodynamics causing embryonic lethality, and could presumably affect placental and cardiac growth (Conway et al., 2002; Lucitti et al., 2007; Udan et al., 2012, 2013; Garcia and Larina, 2014); a YS contribution to placental defects is therefore plausible. However, even though YS and placental defects are observed in this study to occur earlier than reported cardiac phenotypes, we cannot definitively determine at this time whether YS defects are a primary contributing factor to both placental and embryonic phenotypes, or whether, like the placenta, they are secondary to embryonic defects.

The fetal circulatory system connects the developing placenta and heart, and in the mouse these organs sit at opposite ends of a system that also includes an extensive extraembryonic YS vasculature. It is evident, when *Cxadr* is deleted from the mouse genome, a broader vascular defect emerges with embryonic, placental, and YS fetal vessels affected. The role of the vasculature is currently under investigation. While we cannot at this time determine with certainty whether YS defects or embryonic vascular/lymphatic or cardiac defects are the driving force behind the secondary placental abnormalities, Tg^*Tnnt2*-Cre^ and Tg*^Sox2-Cre^* mediated deletion of *Cxadr* nevertheless clearly shows that severe secondary placental defects are the result of a loss of *Cxadr* in non-trophoblast cells and that impaired placental function most likely impacts embryonic viability.

## Conclusion

**Figure 7:**
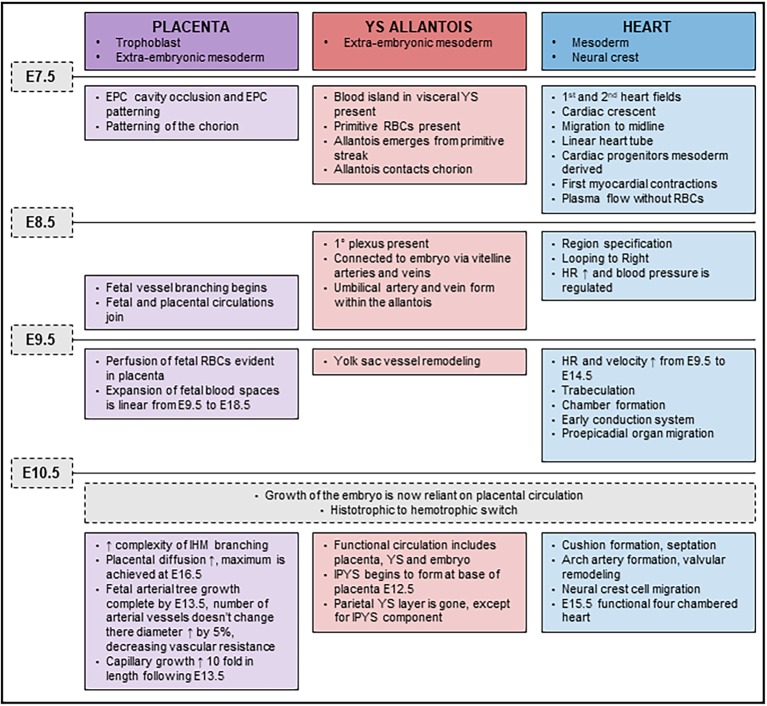
Timeline of placental, yolk sac (YS), and cardiac developmental milestones occurring between E7.5 and E11.5.

While it is not disputed that loss of *Cxadr* impacts cardiac development and function, the notion that the accompanying placental or YS abnormalities could have a compounding impact on the outcome of pregnancy is instead introduced by the current study. We propose that defects observed in the placenta, YS and heart, emerge at a time when development of these systems becomes interdependent (major developmental milestones outlined in Figure 7). By E9.5, once fetal, placental and YS circulations are joined, the importance of optimal function of each component of the system is critical for the continued healthy expansion of embryonic and extraembryonic vascular beds as major remodeling occurs. The rising demands made by the fetus on diffusional exchange (oxygen and carbon dioxide) and placental transfer of nutrients is exponential beyond E10.5 and requires synchronization in development between maternal, placental, and fetal blood flows (Figure 7). The embryonic heart needs to sustain the growing embryo and work hard enough to sustain an expanding extraembryonic vascular system. An inability to maintain endothelial differentiation and sustain branching and IHM patterning to provide adequate surface area for transplacental passage in the *Cxadr* null placenta from E10.5 onwards in all likelihood contributes to the exacerbated heart defects and embryonic lethality. Equally, and most confounding in this scenario, placental and cardiac hemodynamic transactions play a role in determining the subsequent morphology and functional capacity of each other. The conditional deletion of *Cxadr* in the current study shows that significant placental phenotypes can be secondary to primary defects in either YS and/or embryonic structures. Unfortunately, however, definitive conclusions about the precise origin of the extraplacental phenotypes that drive defects in placental growth could not be directly assessed in this study due to the non-selective nature of the Cre mouse lines at our disposal, only that the placental defects observed in *Cxadr* mutants are secondary in nature. What does become evident from this work is that placental development, cardiac development, and vascular function cannot be easily separated and that future studies must be expanded to include an extensive YS vascular plexus.

## Data Availability

All datasets generated for this study are included in the manuscript and/or the Supplementary Files.

## Ethics Statement

Animal experiments conducted in this study were approved by the University of Queensland Animal Ethics Committee and conformed to their guidelines.

## Author Contributions

JO collected and evaluated the data. JP performed the flow cytometry and analysis. JO and DS drafted the manuscript. All authors approved the final version of the manuscript.

### Conflict of Interest Statement

The authors declare that the research was conducted in the absence of any commercial or financial relationships that could be construed as a potential conflict of interest.
