# Supplementary material for: Secondary Placental Defects in Cxadr Mutant Mice
Source: Front Physiol. 2019 May 29;10:622. doi: 10.3389/fphys.2019.00622 (PMC6628872; doi:10.3389/fphys.2019.00622)
Supplement: Supplementary file 1 [file Data_Sheet_1.PDF]

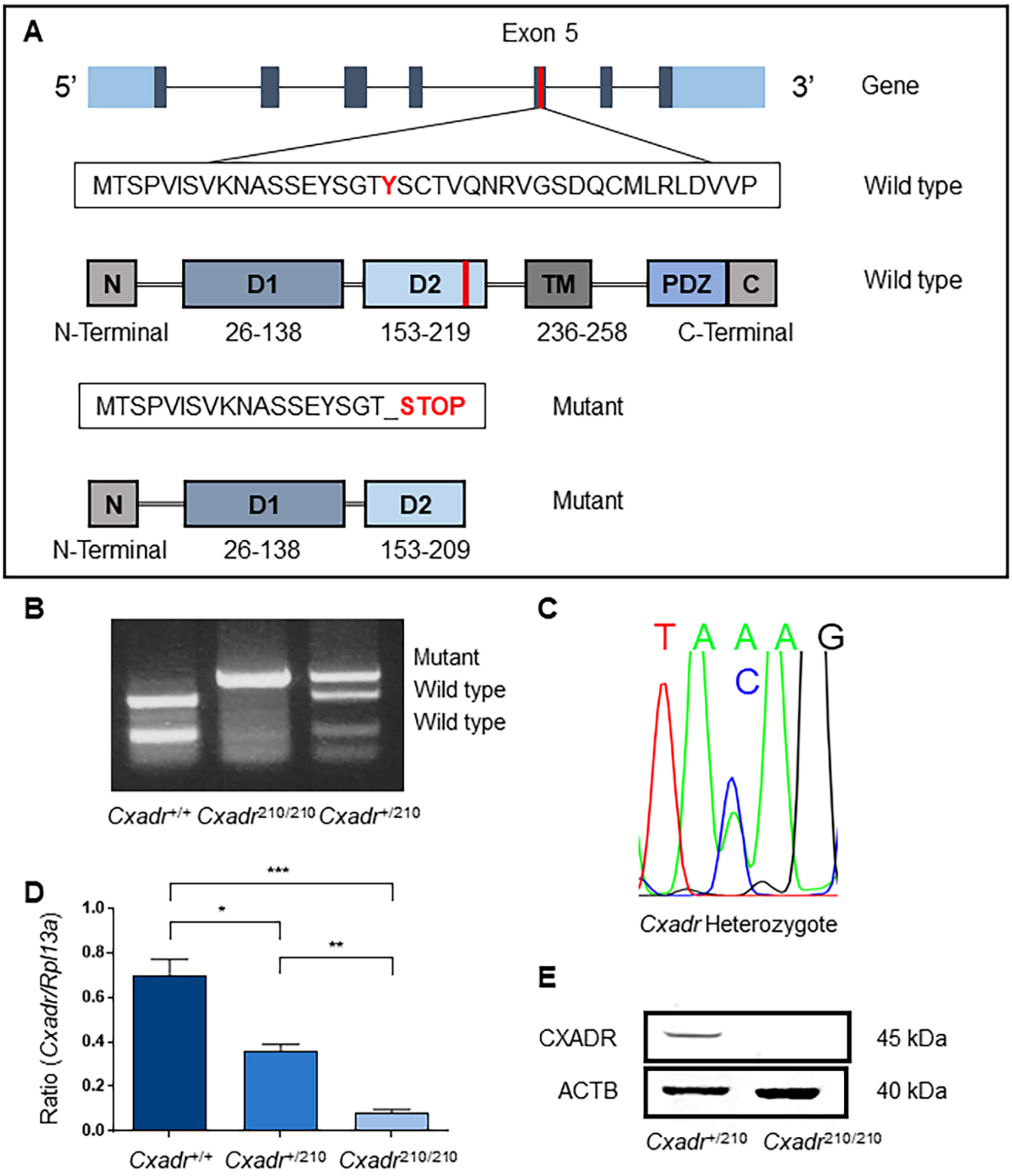

Figure S1 | (A) Random germ line ENU mutagenesis results in a single nucleotide polymorphism at amino acid position 210 in D2 of the CXADR protein. The creation of a premature stop codon, disrupting the Cxadr mRNA, results in a failure to produce the Cxadr mRNA and protein. **(B)** CXADR null pups are homozygous for a non-synonymous substitution (C to A) that results in the loss of a PvuII restriction site. Following heterozygous intercrosses PCR amplification using primers spanning exon 5, followed by PvuII digest was used to genotype embryos and placentas. **(C)** Sequence trace results for Cxadr$^{+/210}$ mice. **(D-E)** Quantitative RT-PCR **(D)** and western blot analysis **(E)** reveals the introduction of a premature stop codon results in a loss of *Cxadr* transcripts and protein in E11.5 mutant embryos. Data represent the mean±SEM. *P<0.05, **P<0.01, ***P<0.001.

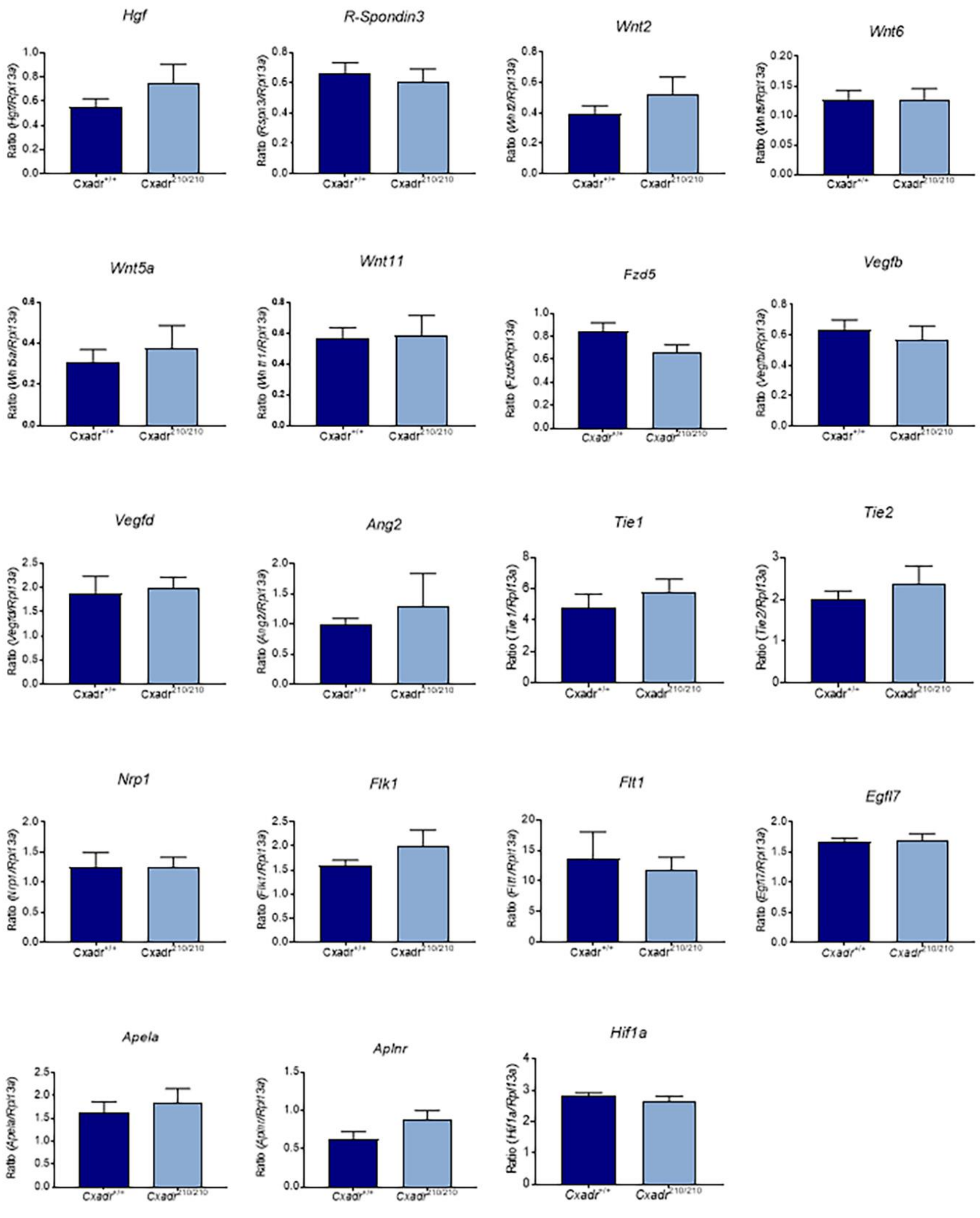

Figure S2 | Quantitative RT-PCR of various growth and angiogenic factors expressed in the allantois and placenta plus their cognate receptors expressed in fetal endothelial cells at E11.5 in *Cxadr*$^{+/+}$ versus *Cxadr* null placentas.

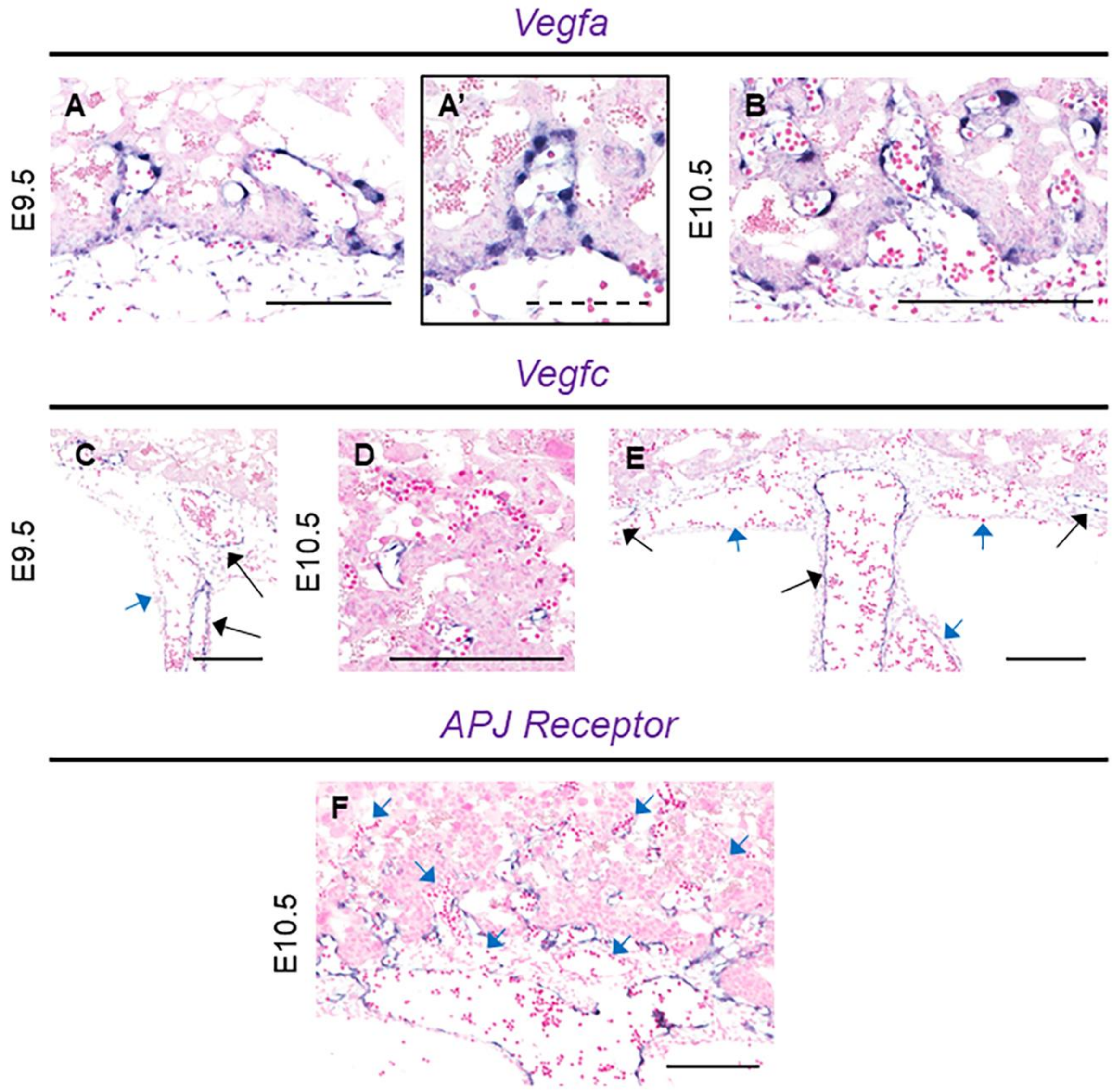

Figure S3 | *In situ* hybridisation of *Vegfa* **(A-B)** and *Vegfc* **(C-E)** at E9.5 and 10.5 in wild type placental samples. **(A-A')** *Vegfa* is expressed in trophoblast cells of the basal chorion layer and in trophoblast cells adjacent to fetal blood vessels. **(B)** *Vegfa* expression is also noted in fetal endothelial cells in the allantois and labyrinth. **(C)** At E9.5 *Vegfc* is expressed in some allantoic vessels (black arrow) and is absent from other vessels (blue arrow). **(D-E)** At E10.5 *Vegfc* is present in some allantoic and labyrinth fetal endothelial vessels. **(F)** Similarly, APJ receptor expression is absent from some endothelial vessels. Scale bars: 100 µm (dashed), 200 µm (solid).

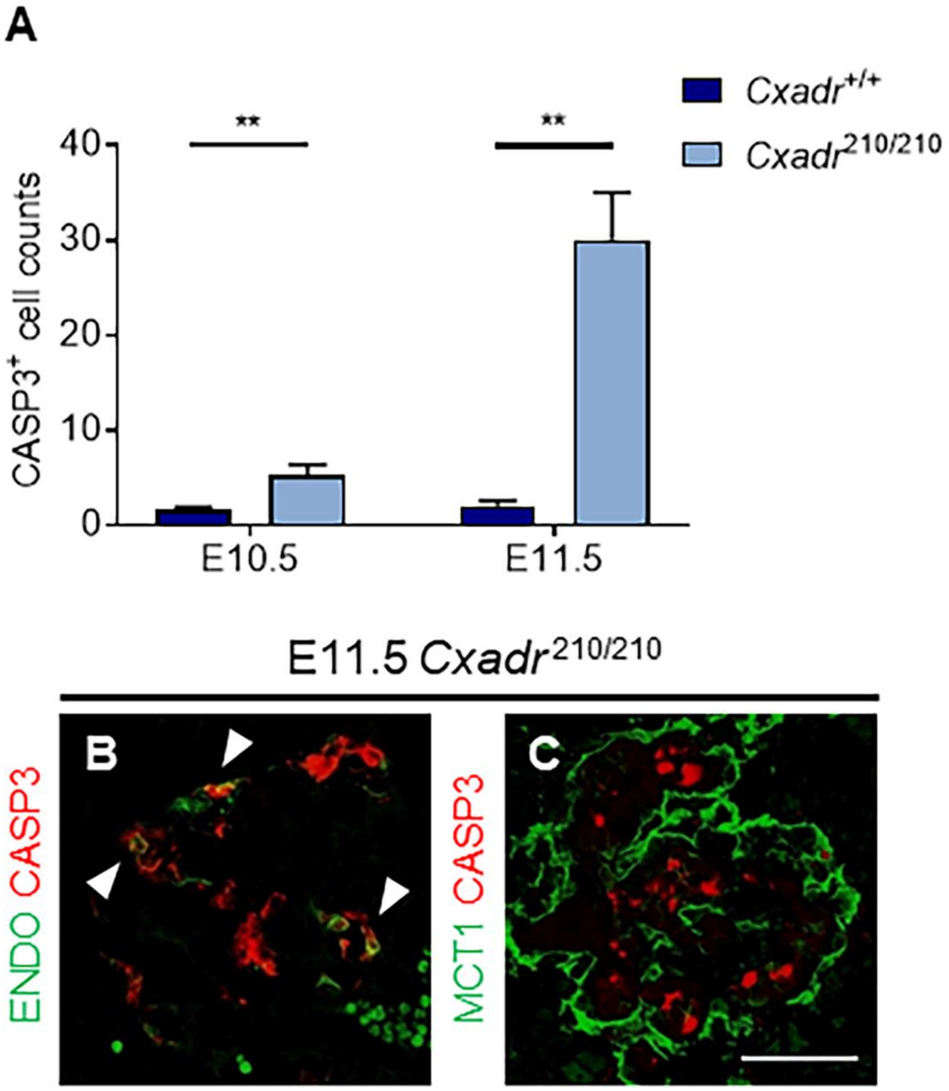

Figure S4 | (A) Quantification of CASP3 positive labyrinth and allantoic cells at E10.5 and E11.5 in wild type and *Cxadr* null placentas. **(B)** Double immunofluorescence for ENDOMUCIN, marking fetal endothelial cells, and CASP3 at E11.5 on *Cxadr* null sections reveals areas of co-localisation (arrowheads). **(C)** Alternatively, double immunofluorescence for MCT1, marking syncytiotrophoblast layer I, and CASP3 show no co-localisation at E11.5. Data represent the mean±SEM **P<0.01. Scale bars: 50µm.

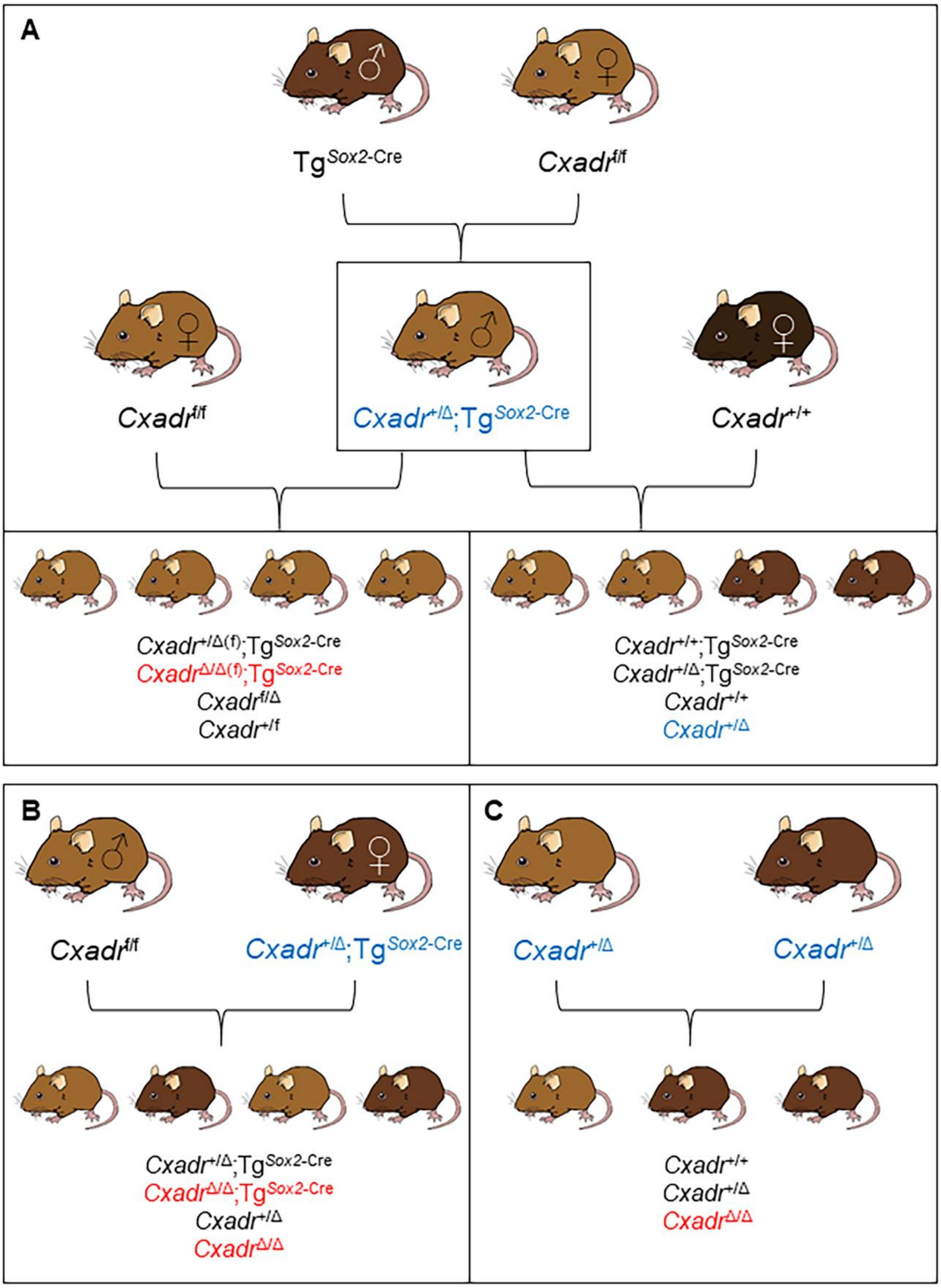

Figure S5 | Tg*Sox2*-Cre breeding strategy: Desired double transgenic animals are achieved by crossing Tg*Sox2*-Cre and *Cxadr*$^{f/f}$ breeding pairs. **(A)** Conditional deletion in the embryo is achieved with paternal inheritance of the *Sox2*-Cre transgene. **(B-C)** Maternal inheritance or heterozygous *Cxadr*$^{+/\Delta}$ crosses (where *Sox2*-Cre has been selectively breed out creating *Cxadr*$^{+/\Delta}$ animals negative for Tg*Sox2*-Cre) are used to derive global knockout embryos and placentas.

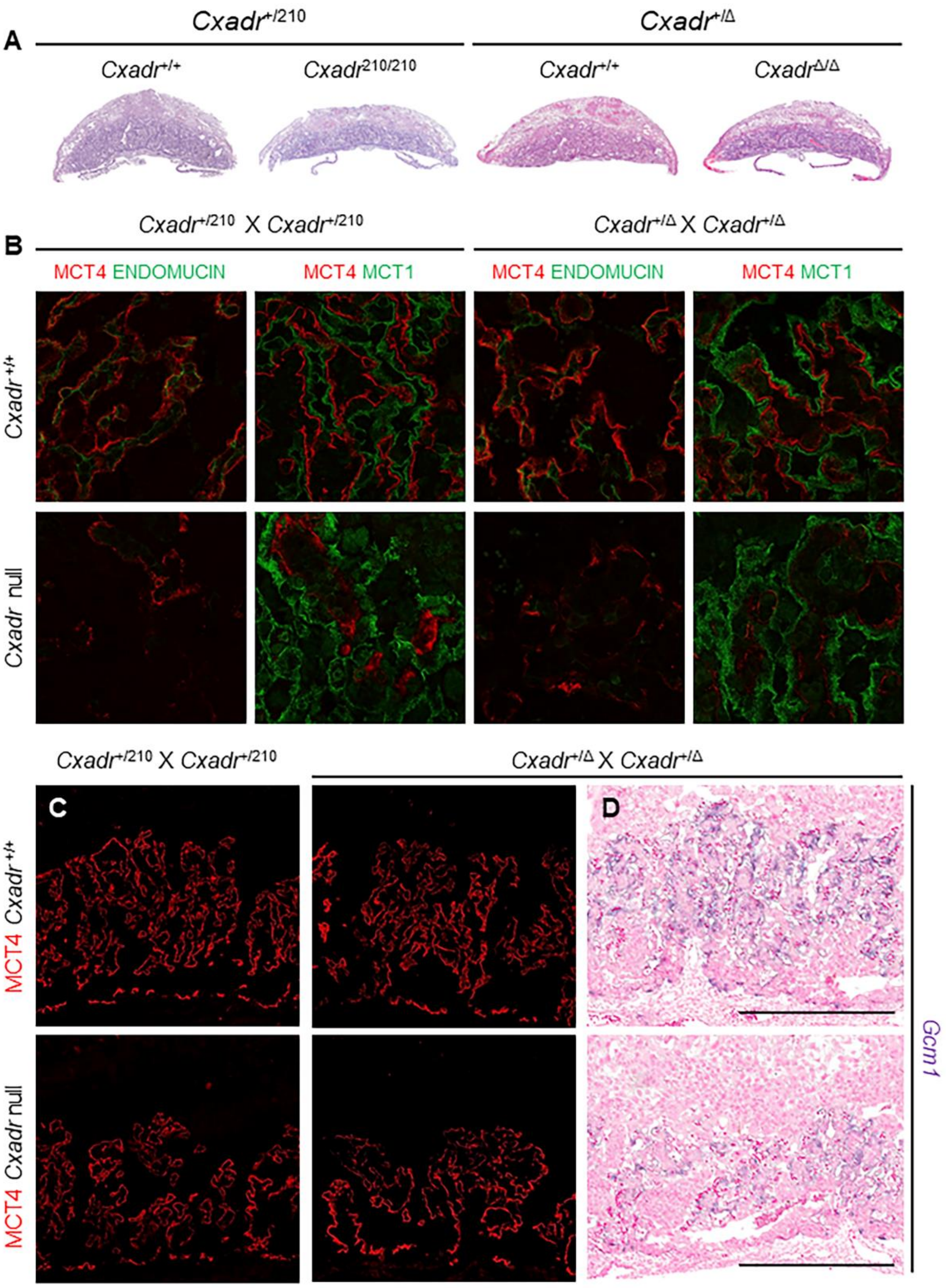

Figure S6 | (A) Histological comparison between *Cxadr*ENU (*Cxadr*$^{+/+}$, *Cxadr*$^{210/210}$) and *Cxadr*Del (*Cxadr*$^{+/+}$, *Cxadr*$^{\Delta/\Delta}$) Hematoxylin & Eosin stained midline placental sections at E11.5. **(B)** Double immunofluorescence (IF) comparing expression of MCT4 (marking syncytial layer II) and ENDOMUCIN (marking fetal endothelial cells) plus MCT4 and MCT1 (marking syncytial layer I) between *Cxadr*ENU (*Cxadr*$^{+/+}$, *Cxadr*$^{210/210}$) and *Cxadr*Del (*Cxadr*$^{+/+}$, *Cxadr*$^{\Delta/\Delta}$) in placental sections at E11.5. **(C)** IF comparing expression of MCT4 between *Cxadr*ENU (*Cxadr*$^{+/+}$, *Cxadr*$^{210/210}$) and *Cxadr*Del (*Cxadr*$^{+/+}$, *Cxadr*$^{\Delta/\Delta}$) in placental sections at E11.5. **(D)** *In situ* hybridisation for *Gcm1* (marking syncytial layer II precursor cells) at E11.5 in *Cxadr*$^{+/+}$ and *Cxadr*$^{\Delta/\Delta}$ labyrinth samples. Scale bars: 500 µm.

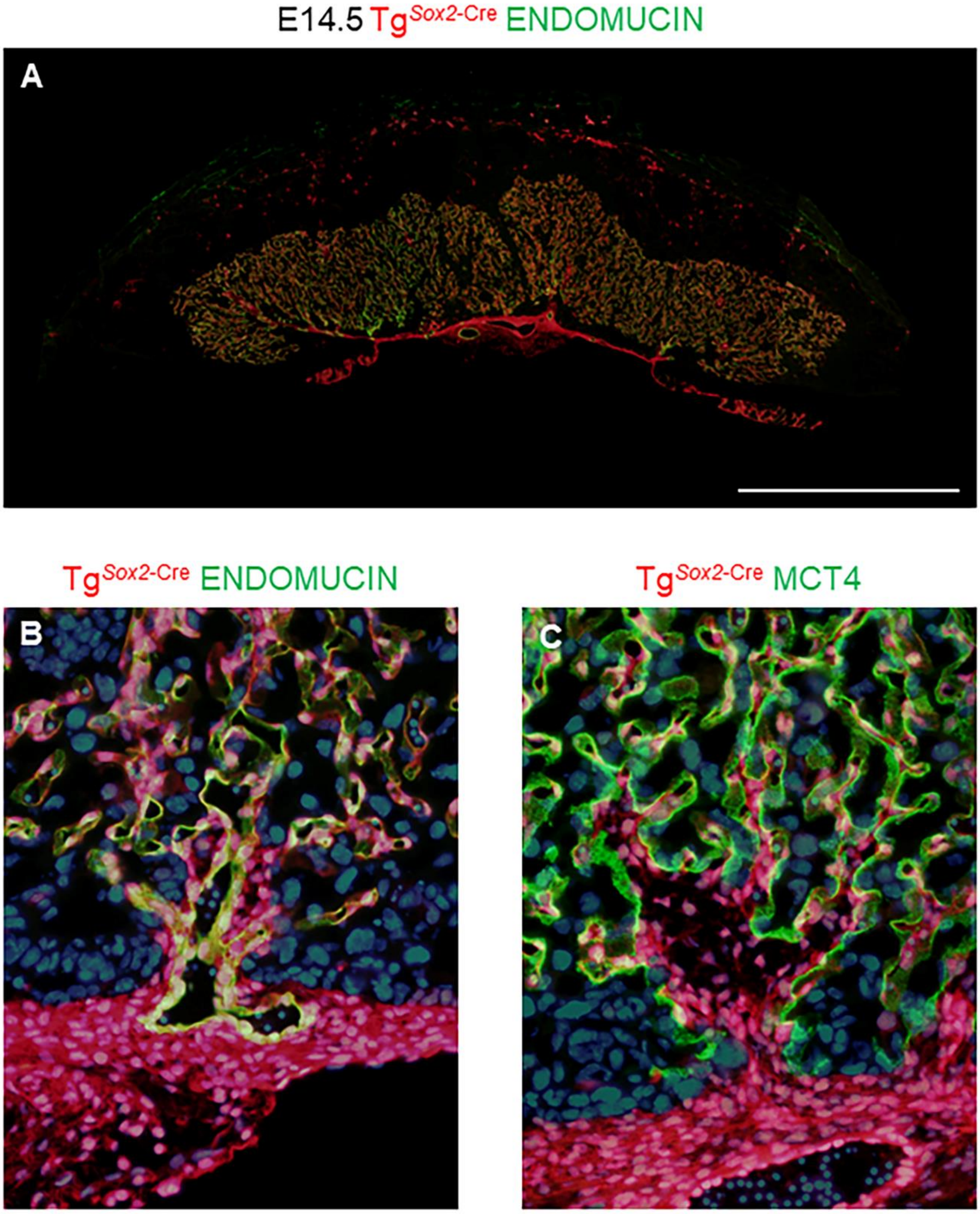

Figure S7 | **(A)** Tg*Sox2*-Cre driven expression of tdTomato (DsRed) coupled with immunofluorescence (IF) for ENDOMUCIN (green), marking fetal endothelial cells, at E14.5 in midline placental section. **(B-C)** Higher magnification shows Tg*Sox2*-Cre driven expression of tdTomato coupled with IF for ENDOMUCIN **(B)** and Tg*Sox2*-Cre driven expression of tdTomato coupled with IF for MCT4 (green) **(C)**, marking syncytial layer II. Scale bar: 2mm.

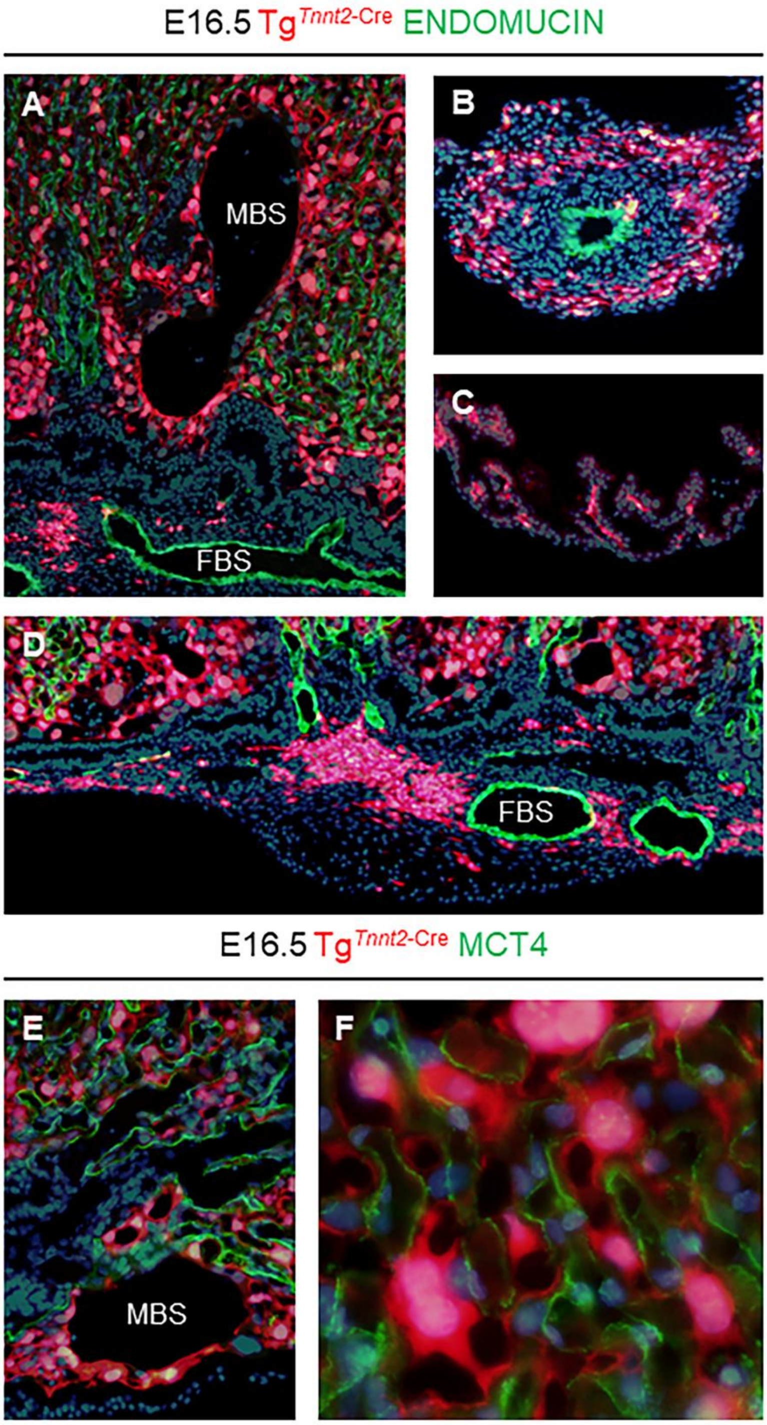

Figure S8 | (A-D) Frozen midline placental cross-sections show Tg$^{\textit{Tnnt2}\text{-Cre}}$ (tdTomato) expression coupled with immunofluorescence for ENDOMUCIN, marking fetal endothelial cells, at E16.5 in Tg$^{\textit{Tnnt2}\text{-Cre}}$;Tg^{tdTom} placentas. TdTomato positive cells, that do not co-localise with ENDOMUCIN, are seen surrounding maternal blood spaces (MBSs) in the labyrinth **(A)** (likely marking sinusoidal TGCs), in umbilical vessels **(B)**, in the yolk sac **(C)** and in the allantois **(A, D)**. **(E-F)** Double immunofluorescent images reveal MCT4 positive syncytiotrophoblast II cells do not co-localise with tdTomato (Tg$^{\textit{Tnnt2}\text{-Cre}}$) expression. **(F)** Higher magnification of E16.5 labyrinth.

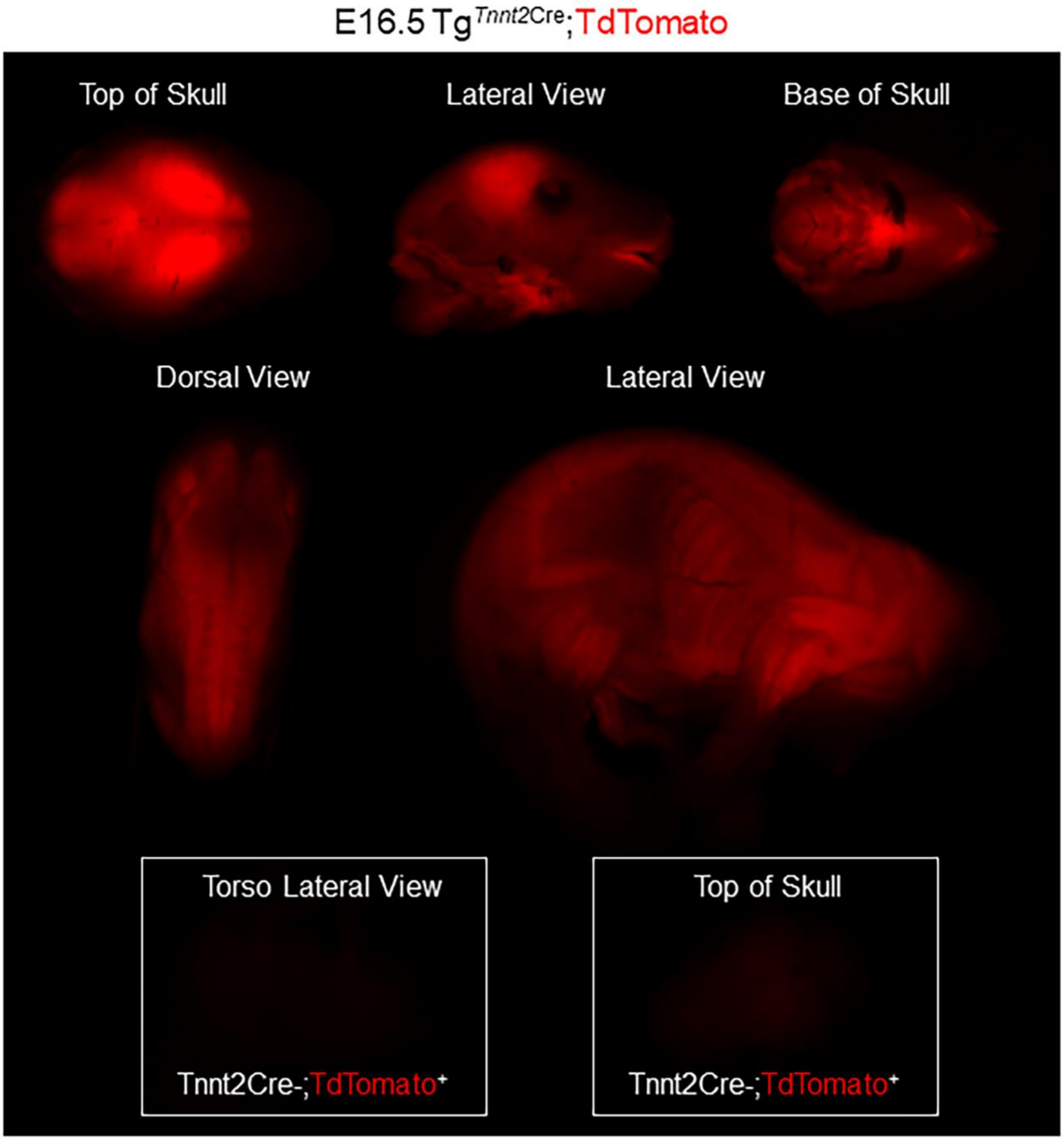

Figure S9 | Whole mount images depicting Tg$^{\mathit{Tnnt2}\text{-Cre}}$ expression in E16.5 embryos revealed by synthesis of tdTomato (DsRed fluorescent protein) transcripts. Control animals (Tg$^{\mathit{Tnnt2}\text{-Cre}}$ negative; Tg^{tdTom} positive) show no fluorescent expression (insets).

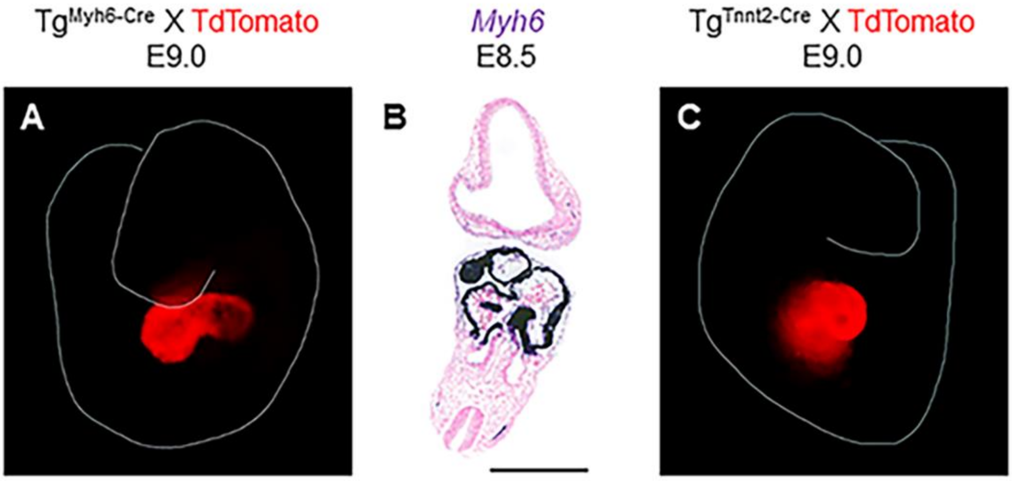

Figure S10 | Heart specific transgene expression. **(A)** Whole mount image depicting Tg$^{\textit{Myh6}\text{-Cre}}$ expression in E9.0 developing heart, revealed by synthesis of tdTomato (DsRed fluorescent protein) transcripts in Tg$^{\textit{Myh6}\text{-Cre}}$ positive;Tg^{tdTom} positive animals. **(B)** *In situ* hybridization for *Myh6* is seen in the developing heart of wild type E8.5 embryos. **(C)** Whole mount image depicting Tg$^{\textit{Tnnt2}\text{-Cre}}$ expression in E9.0 developing heart, revealed by synthesis of tdTomato (DsRed fluorescent protein) transcripts in Tg$^{\textit{Tnnt2}\text{-Cre}}$ positive;Tg^{tdTom} positive animals.
