# Supplementary material for: Secondary Placental Defects in Cxadr Mutant Mice
Source: Front Physiol. 2019 May 29;10:622. doi: 10.3389/fphys.2019.00622 (PMC6628872; doi:10.3389/fphys.2019.00622)
Supplement: Supplementary file 2 [file Data_Sheet_2.PDF]

Table S1: CXADR interactions

Protein/Virus	Function	Reference
**Extracellular/Intercellular**		
**CXADR**	Cell adhesion and recognition.	(Cohen et al., 2001a; Honda et al., 2000; Hotta et al., 2003)
**Adenovirus (Subgroups A/C/D/E/F)**	Viral receptor.	(Leon et al., 1998)
**Coxsackie virus (CVB1-CVB6)**	Viral receptor.	(Chung et al., 2005; Coyne and Bergelson, 2006; Triantafilou and Triantafilou, 2004)
**JAM-L (Amica1)**	Cell recognition.	(Guo et al., 2008; Zen et al., 2005)
**JAM-C (JAM3)**	Spermatid differentiation.	(Mirza et al., 2006)
**Laminin-1**	Neurite outgrowth.	(Patzke et al., 2010)
**Fibronectin**	Neurite outgrowth.	(Patzke et al., 2010)
**Claudin-3**	Tight/Paracellular junction dynamics.	(Mirza et al., 2007)
**Occludin**	Tight junction integrity.	(Mirza et al., 2007)
**N-Cadherin**	Cell adhesion.	(Wang et al., 2007)
**Tenascin-R**	Unknown.	(Matthaus et al., 2017)
**Agrin**	Unknown.	(Matthaus et al., 2017)
**Intracellular**		
**TJP1/ZO-1**	Epithelial tight junction dynamics.	(Cohen et al., 2001a; Cohen et al., 2001b; Lim et al., 2008; Shono et al., 2007; Wang et al., 2007)
**MAGI-1**	Localization of proteins at the membrane.	(Excoffon et al., 2004; Kolawole et al., 2012)
**E-Cadherin**	Epithelial junction integrity.	(Hussain et al., 2011; Morton et al., 2013)
**PICK1**	Localization of proteins at the membrane.	(Excoffon et al., 2004)
**PSD-95**	Clustering proteins at the membrane.	(Excoffon et al., 2004; Excoffon et al., 2012)
**ASIC3**	Clustering ion channels at the membrane.	(Excoffon et al., 2012)
**LNX/LNX2**	Localization of proteins at specific cell sites.	(Mirza et al., 2005; Sollerbrant, 2002)
**Cx45**	Localization of Cx45 to specific sites of the membrane.	(Lim et al., 2008; Lisewski et al., 2008)
**Harmonin**	Not determined.	(Excoffon et al., 2006)
**MUPP1**	Tight junction integrity.	(Coyne et al., 2004)
**Actin and dynamin**	Regulation of cytoskeleton proteins.<br>Potential endocytosis.	(Huang et al., 2007; Salinas et al., 2014)
**Src**	Localization of E-cadherin at the membrane.	(Morton et al., 2013)
**Cell Signalling**		
**PI3K and Akt**	Cell signalling<br>Co-receptor.	(Verdino and Wilson, 2011; Verdino et al., 2011)
**ROCK**	Cell adhesion.	(Saito et al., 2013)
**β-catenin**	Adherens and tight junction dynamics.	(Lim et al., 2008; Walters et al., 2002)
**PKCδ**	Epithelial homeostasis.	(Morton et al., 2013)
**P21 and Rb**	Contact initiated growth inhibition.	(Okegawa et al., 2001)
**p44/42 MAPK**	Localization of proteins at the membrane.	(Farmer et al., 2009)
**Raf-MEK-ERK (MAPK/ERK)**	Regulation of cell adhesion molecules.<br>Contact initiated growth inhibition.	(Anders et al., 2003; Huang et al., 2005)
**Internalisation**		
**Clathrin/dynamin**	CVB3 internalization.	(Chung et al., 2005)

**Caveolin-1/DAF**	CVB internalization.	(Coyne and Bergelson, 2006)
**Lipid rafts and lipid-rich microdomains**	Endocytosis.	(Excoffon et al., 2003; Salinas et al., 2014; Triantafilou and Triantafilou, 2004)
**Integrin**	Adenovirus internalization.	(Farmer et al., 2009; Venkatraman et al., 2005)
	**Upstream factors**	
**FSH**	Sertoli cell maturation and function.	(Mirza et al., 2007)
**Estrogen**	Estrogen receptor.	(Vindrieux et al., 2011)
**TNF-α and IFN-γ**	Cell adhesion molecule.	(Vincent et al., 2004)
**Tfap2c**	TS regulatory network.	(Kidder and Palmer, 2010)
**Eomes**	TS regulatory network.	(Kidder and Palmer, 2010)
	**Epithelial-mesenchymal transition**	
**Zeb-1**	Cell adhesion.	(Lacher et al., 2011)
**TGF-β**	Cell adhesion.	(Lacher et al., 2006)
**SNAIL1-SMAD3/4**	Cell adhesion.	(Vincent et al., 2009)

Table S2: CXADR expression

Organ	Expression	Reference
**Bladder**	Late gestation: Uroepithelium<br>Postnatal: Between uroepithelium basal cells and basal lamina	(Gye et al., 2011)
**Blood**	Hematopoietic progenitors (10-15%), differentiated erythroid and myeloid cells	(Rebel et al., 2000)
**Bone marrow**	Mesenchymal stem cells	(Hung et al., 2004)
**Cardiac**	E9: primitive left ventricle, weak expression seen in right-sided heart structures.<br>E11.5: Ventricle myocardium<br>Postnatal: Intercalated discs of the AV node	(Asher et al., 2005; Chen et al., 2006; Dorner et al., 2005; Lim et al., 2008; Lisewski et al., 2008)
**Cochlear**	Neonatal (P1-15): Cochlear cell types<br>Postnatal: Restricted to pillar and strial cells	(Excoffon et al., 2006)
**Epithelial cells**	Postnatal: Colonic mucosa	(Zen et al., 2005)
**Endothelial cells**	Microvascular endothelial cells<br>Umbilical vein endothelial cells	(Guo et al., 2008; Vincent et al., 2004)
**Kidney**	Pronephros	(Raschperger et al., 2008)
**Liver**	Hepatocytes	(Liu et al., 2013)
**Lungs**	Alveolar epithelial cells	(Sun et al., 2012)
**Lymphatics**	E14.5 – E16.5: Lymphatic endothelial vessels	(Mirza et al., 2012)
**Mesoderm**	Mesoderm	(Tashiro et al., 2015)
**Nervous system**	E8.5 – P7: Neural tube/neuroepithelium<br>E9.5 – E11.5: Cranial motor nerves<br>E13.5 – P7: Optic nerve, motor nerves<br>Postnatal: Few cells at sites of neurogenesis (Hippo/SVZ/RMZ)	(Hauwel et al., 2005; Honda et al., 2000; Hotta et al., 2003)
**Olfactory system**	Embryonic: Olfactory sensory neurons, olfactory bulb and olfactory epithelium<br>Postnatal: Olfactory epithelium	(Venkatraman et al., 2005)
**Oropharynx**	Basal layer of oropharyngeal epithelium	(Hutchin et al., 2000)
**Pancreas, Thymus and Intestines**	Embryonic: Intestinal epithelium; scattered throughout pancreas; thymic epithelium<br>Postnatal: Expression in epithelium of all 3 organs, reticular cells of the thymus	(Kallewaard et al., 2009; Pazirandeh et al., 2011; Tomko et al., 2000)
**Pituitary**	E11.5: Oral epithelium<br>E13.5 and postnatal: Marginal cell layer and parenchyma	(Chen et al., 2013)
**Preimplantation embryo**	All cells from mature human oocytes through compaction and blastocyst stages.<br>Trophectoderm and Inner cell mass.<br>Undifferentiated human and mouse ESCs	(Kawabata et al., 2005; Krivega et al., 2014; Kwon et al., 2016; Oh et al., 2016)
**Skin**	Keratinocytes	(Deng et al., 2013; Witherden et al., 2010)
**Testis**	Embryonic: Sertoli and germ cells<br>Postnatal: Spermatogonia, preleptotene spermatocyte, spermatids	(Mirza et al., 2007; Sultana et al., 2014; Wang et al., 2007) ((Su et al., 2012)

Table S3: Genotyping primers

Transgene	Forward	Reverse	Amplicon size (bps)
CxadrENU	CTGCAGGCTGGTTGTATGAA	AAGCAAACACACCACACAGG	WT: 562 and 293 (post RE digest), Null: 801
Cxadr Flox	GAGACTGGATTATGAGTTCCAGG CTTTAG	CCTGCTCCAGATTCCCACAATTCC	WT: 741, Flox: 874, Null: 409
Cre	TGCATGATCTCCGGTATTGA	CGTACTGACGGTGGGAGAAT	400
TdTom	AAGGGAGCTGCAGTGGAGTA<br>GGCATTAAAGCAGCGTATCC	CCGAAAATCTGTGGGAAGTC<br>CTGTTCCTGTACGGCATGG	WT: 297, Mutant: 196

Table S4: Mouse lines

Mouse Strain	Reference
*Cxadr*ENU:210$^{\text{Y-Stop}}$	Australian Phenomics Facility<br>Missense Mutation Library
*Cxadr*Flox (B6;129S2-Cxadr$^{\text{tm1.1lcs}}$/J)	Pazirandeh et al., 2011
Sox2-Cre (Tg[Sox2-cre]1Amc/J)	Hayashi et al ., 2002; Hayashi et al., 2003
Myh6-Cre (B6.FVB-Tg[*Myh6*-cre]2182Mds/J)	Agah et al., 1997
Tnnt2-Cre (Tg[*Tnnt2*-cre]5Blh/JiaoJ)	Wang et al., 2000; Wang et al., 2001
TdTomato (B6;Cg-*Gt[ROSA]26Sor*$^{\textit{tm9(CAG-tdTomato)Hze}}$/J)	Madisen et al., 2009

Table S5: qRT-PCR primers

Gene	Forward	Reverse
Ang1	CAGCTTTGGAATCTCTGTTG	GCTTCTTCTCTTCATCATACG
Ang2	CTCCAGGTACACAAAATTCC	TTTCACCATCATACTTTCGC
Apela	AAGAAAAGATGCGATTCCAG	GGGAAAGTTAACTGGTTTCTG
Apln	GCCTTTCTAAAGCAGGATTG	AATAGATGTGAGGGTTCCAG
Aplnr	TACTTCTTCATTGCCCAAAC	GAAGATGTCAAAGTCACAGG
cMet	CGACAAATACGTTGAAATGC	GATCTACATAGGAGAATGCAC
Ctsq	TTCATTGGCCCAATACCCTA	GAAAGCTCCCAGAATTCACA
Cxadr	CATCATGATATCAGGGAAGAT	AATACCCCTCCATGTTAGAG
Egfl7	GATGTGCTAGAACAGAAACTG	CTCAGTGAATCAATTCGGTC
Fzd5	GATCCTCCGAGAGTTCTG	ACCTGTTGGTTTCTTTTTCTC
Gcm1	ATCTTTTTCCAGTCCAAAGG	CACTTTCTTCATGGCTCTTC
Hgf	CAAATGCAAGGACCTTAGAG	CTTGTTTTGGATAAGTTGCC
Hif1a	CGATGACACAGAAACTGAAG	GAAGGTAAAGGAGACATTGC
Nrp1	TTATCTTTCAGGGAAACACC	TCCAGAGCAAGGATAATCTG
Rhox4b	GTTGAATGGTGGGAAGACA	TTTGTCCCATTCCACTGCT
Rpl13a	CCTATGACAAGAAAAAGCGG	CAGGTAAGCAAACTTTCTGG
R-spondin3	CCAAGTGGATATTACGGAAC	CCATAGTATGATTGTTGGCTTC
Syna	ATGGAGAAACCCCTTACGCT	TAGGGGTCTTTGTGTCCCTG
Tie1	AGAGGTGGAGTTCAACATAG	TTTGGTAGACAAAAGCATGG
Tie2 (Tek)	ATTTCCGTCAAAGTTCTTCC	AAGCTTCTTGGATTTGATGG
Vegfa	TAGAGTACATCTTCAAGCCG	TCTTTCTTTGGTCTGCATTC
Vegfb	GATCCTCATGATCCAGTACC	TTTGGTCTGCATTCACATTG
Vegfc	ACATGCAGTTGTTACAGAAG	GCATAGACAGTGCTAATGTG
Vegfd (Figf)	CTCTTTGAGATATCAGTGCC	GAGGACATTCATCTTCTTCTG
Vegfr1 (Flt1)	TTGTAAACGTGAAACCTCAG	GATTCTTCATTCTCAGTGCAG
Vegfr2 (Flk1, Kdr)	AATGGTACAGAAATGGAAGG	GCATCTCTTTCAGTCACTTC
Wnt2	GTTAATATGAACGTCCCTCTC	TCATGTACCACCATGAAGAG
Wnt6	TAGTAGTGGGCTTAGTTGTC	TTTTACAGTTCATGACGAGC
Wnt5a	AATTCTTGGTGGTCTCTAGG	CAGAGTTTCTTCTGTCCTTG
Wnt11	CCAATAAACTGATGCGTCTAC	ATTTACACTTCGTTTCCAGG

Table S6: In situ hybridisation primers

Gene	Forward (T3)	Reverse (T7)
Aplnr	ACTATGGGGCTGACAACCAG	CAAACTGCCCAGCATGTAGA
cMet	CTACACCCCAGCCCAAACTA	CTCAGGCAGATTCCCAAGAG
Ctsq	AATTAACCCTCACTAAAGGG	TAATACGACTCACTATAGGG
Cxadr	ACCAGGGACCACTGGACA	GGCGCACGTTCAAAGTCT
Gcm1	AATTAACCCTCACTAAAGGG	TAATACGACTCACTATAGGG
Mest	GAGAGAGTGGTGGGTCCAAG	CGATCACTCGATGGAACCTC
Myh6	GAAAAGGAGGCATTGATTTC	CTTCTCCCAGCTGTTCAGTC
Syna	AATTAACCCTCACTAAAGGG	TAATACGACTCACTATAGGG
Vegfa	CGGAAACTTTTCGTCCAACT	GCGAGTCTGTGTTTTTGCAG
Vegfc	CAAGGCTTTTGAAGGCAAAG	CACAGCGGCATACTTCTTCA

Table S7: Antibodies

Primary Antibody	Animal	Tissue processing	Antigen Retrieval	Concentration	Distributor
MCT4 (AB331I4P)	Rabbit	Frozen/Paraffin	EDTA/Citrate	1:200	Millipore
MCT1 (AB1286-I)	Chicken	Frozen/Paraffin	EDTA/Citrate	1:200	Millipore
CXADR (ab100811)	Rabbit	Frozen/Paraffin	Citrate/None/ Methanol/ Acetone	1:500	Abcam
ENDOMUCIN V.SC7 (SC-53941)	Rat	Frozen/Paraffin	EDTA/Citrate	1:100	Santa Cruz
CASP3 Asp175 (#9661)	Rabbit	Frozen/Paraffin	Citrate	1:300	Cell Signalling
CD31 PECAM1 (ab28364)	Rabbit	Frozen/Paraffin	Citrate	1:100	Abcam
**Secondary Antibody**	**Animal**			**Concentration**	**Distributor**
Anti-Rat 488	Donkey			1:400	Abcam (ab150153)
Anti-Rabbit Cy3	Donkey			1:400	Jackson Immuno Research (711-165-152)
Anti-Rabbit 488	Donkey			1:400	Jackson Immuno Research (711-545-152)
Anti-Chicken 488	Donkey			1:400	Jackson Immuno Research (703-546-155)
Anti-rabbit HRP	Goat			1:500	Invitrogen

Table S8: Genotypes of progeny from *Cxadr*ENU intercrosses

<table>
<tr><th>Stage</th><th>Cxadr$^{+/+}$</th><th>Cxadr$^{+/210}$</th><th colspan="2">Cxadr$^{210/210}$</th><th>Dead (genotype)</th></tr>
<tr><td>E10.5</td><td>23
33.3%</td><td>30
43.5%</td><td colspan="2">14
20.3%</td><td>2 (Cxadr$^{+/+}$)
2.9%</td></tr>
<tr><td>E11.5</td><td>18
31%</td><td>24
41.4%</td><td>11
19%</td><td>4 (↓HR)
6.9%</td><td>1 (Cxadr$^{+/210}$)
1.7%</td></tr>
<tr><td>E12.5</td><td>7
22.6%</td><td>16
51.6%</td><td colspan="2">0
0%</td><td>8 (Cxadr$^{210/210}$)
25.8%</td></tr>
<tr><td>Expected</td><td>25%</td><td>50%</td><td colspan="2">25%</td><td></td></tr>
</table>

↓HR = Erratic heart rate, E = Embryonic day

Table S9: Genotypes of progeny from *Cxadr*$^{+/\Delta}$;Tg$^{Myh6\text{-Cre}}$ crossed to *Cxadr*$^{f/f}$ mice

Stage	*Cxadr* $^{\Delta/\Delta(f)}$;Tg$^{Myh6\text{-Cre}}$	*Cxadr*$^{+/\Delta}$;Tg$^{Myh6\text{-Cre}}$	*Cxadr*$^{+/\Delta}$	*Cxadr*$^{+/f}$
**E11.5**	8	4	9	2
**E12.5**	7	7	3	4
**E13.5**	2	6	2	7
**E16.5**	4 (1)	9	6	9

(1) = Embryo with severe lymphatic oedema

Supplemental References

Anders, M., Christian, C., McMahon, M., McCormick, F. and Korn, W. M. (2003). Inhibition of the Raf/MEK/ERK pathway up-regulates expression of the coxsackievirus and adenovirus receptor in cancer cells. Cancer Res. 63, 2088–2095.

Asher, D. R., Cerny, A. M., Weiler, S. R., Horner, J. W., Keeler, M. L., Neptune, M. A., Jones, S. N., Bronson, R. T., DePinho, R. A. and Finberg, R. W. (2005). Coxsackievirus and adenovirus receptor is essential for cardiomyocyte development. Genesis. 42, 77–85.

Chen, J. W., Zhou, B., Yu, Q. C., Shin, S. J., Jiao, K., Schneider, M. D., Baldwin, H. S. and Bergelson, J. M. (2006). Cardiomyocyte-Specific Deletion of the Coxsackievirus and Adenovirus Receptor Results in Hyperplasia of the Embryonic Left Ventricle and Abnormalities of Sinuatrial Valves. Circulation Research. 98, 923–930.

Chen, M., Kato, T., Higuchi, M., Yoshida, S., Yako, H., Kanno, N. and Kato, Y. (2013). Coxsackievirus and adenovirus receptor-positive cells compose the putative stem/progenitor cell niches in the marginal cell layer and parenchyma of the rat anterior pituitary. Cell Tissue Res. 354, 823–836.

Chung, S.-K., Kim, J.-Y., Kim, I.-B., Park, S.-I., Paek, K.-H. and Nam, J.-H. (2005). Internalization and trafficking mechanisms of coxsackievirus B3 in HeLa cells. Virology. 333, 31–40.

Cohen, C. J. C., Shieh, J. T. J., Pickles, R. J. R., Okegawa, T. T., Hsieh, J. T. J. and Bergelson, J. M. J. (2001a). The coxsackievirus and adenovirus receptor is a transmembrane component of the tight junction. Proc. Natl. Acad. Sci. U.S.A. 98, 15191–15196.

Cohen, C. J., Gaetz, J., Ohman, T. and Bergelson, J. M. (2001b). Multiple Regions within the Coxsackievirus and Adenovirus Receptor Cytoplasmic Domain Are Required for Basolateral Sorting. Journal of Biological Chemistry. 276, 25392–25398.

Coyne, C. B. and Bergelson, J. M. (2006). Virus-Induced Abl and Fyn Kinase Signals Permit Coxsackievirus Entry through Epithelial Tight Junctions. Cell 124, 119–131.

Coyne, C. B., Voelker, T., Pichla, S. L. and Bergelson, J. M. (2004). The Coxsackievirus and Adenovirus Receptor Interacts with the Multi-PDZ Domain Protein-1 (MUPP-1) within the Tight Junction. Journal of Biological Chemistry. 279, 48079–48084.

Deng, X., Jia, C., Chen, F., Liu, J. and Zhou, Z. (2013). Effects of heat stress on the expression of the coxsackievirus and adenovirus receptor in mouse skin keratinocytes. Exp Ther Med. 6(4):1029-1033

Dorner, A. A., Wegmann, F., Butz, S., Wolburg-Buchholz, K., Wolburg, H., Mack, A., Nasdala, I., August, B., Westermann, J. and Rathjen, F. G. (2005). Coxsackievirus-adenovirus receptor (CAR) is essential for early embryonic cardiac development. J. Cell. Sci. 118, 3509–3521.

Excoffon, K. J. D. A., Avenarius, M. R., Hansen, M. R., Kimberling, W. J., Najmabadi, H., Smith, R. J. H. and Zabner, J. (2006). The Coxsackievirus and Adenovirus Receptor: A new

adhesion protein in cochlear development. Hearing Research. 215, 1–9.

Excoffon, K. J. D. A., Hruska-Hageman, A., Klotz, M., Traver, G. L. and Zabner, J. (2004). A role for the PDZ-binding domain of the coxsackie B virus and adenovirus receptor (CAR) in cell adhesion and growth. J. Cell. Sci. 117, 4401–4409.

Excoffon, K. J. D. A., Kolawole, A. O., Kusama, N., Gansemer, N. D., Sharma, P., Hruska-Hageman, A., Petroff, E. and Benson, C. J. (2012). Coxsackievirus and adenovirus receptor (CAR) mediates trafficking of acid sensing ion channel 3 (ASIC3) via PSD-95. Biochemical and Biophysical Research Communications. 425, 13–18.

Excoffon, K. J. D. A., Moninger, T. and Zabner, J. (2003). The Coxsackie B Virus and Adenovirus Receptor Resides in a Distinct Membrane Microdomain. Journal of Virology. 77, 2559–2567.

Farmer, C., Morton, P. E., Snippe, M., Santis, G. and Parsons, M. (2009). Coxsackie adenovirus receptor (CAR) regulates integrin function through activation of p44/42 MAPK. Experimental Cell Research. 315, 2637–2647.

Guo, Y. L., Bai, R., Chen, C. X.-J., Liu, D. Q., Liu, Y., Zhang, C. Y. and Zen, K. (2008). Role of Junctional Adhesion Molecule-Like Protein in Mediating Monocyte Transendothelial Migration. Arteriosclerosis, Thrombosis, and Vascular Biology. 29, 75–83.

Hauwel, M., Furon, E. and Gasque, P. (2005). Molecular and cellular insights into the coxsackie–adenovirus receptor: role in cellular interactions in the stem cell niche. Brain Research Reviews. 48, 265–272.

Honda, T. T., Saitoh, H. H., Masuko, M. M., Katagiri-Abe, T. T., Tominaga, K. K., Kozakai, I. I., Kobayashi, K. K., Kumanishi, T. T., Watanabe, Y. G. Y., Odani, S. S., et al. (2000). The coxsackievirus-adenovirus receptor protein as a cell adhesion molecule in the developing mouse brain. Brain Res. Mol. Brain Res. 77, 19–28.

Hotta, Y., Honda, T., Naito, M. and Kuwano, R. (2003). Developmental distribution of coxsackie virus and adenovirus receptor localized in the nervous system. Brain Res. Dev. Brain Res. 143, 1–13.

Huang, K.-C., Altinoz, M., Wosik, K., Larochelle, N., Koty, Z., Zhu, L., Holland, P. C. and Nalbantoglu, J. (2005). Impact of the coxsackie and adenovirus receptor (CAR) on glioma cell growth and invasion: Requirement for the C-terminal domain. Int. J. Cancer. 113, 738–745.

Huang, K.-C., Yasruel, Z., Guérin, C., Holland, P. C. and Nalbantoglu, J. (2007). Interaction of the Coxsackie and adenovirus receptor (CAR) with the cytoskeleton: Binding to actin. FEBS Letters. 581, 2702–2708.

Hung, S.-C., Lu, C.-Y., Shyue, S.-K., Liu, H.-C. and Ho, L. L.-T. (2004). Lineage differentiation-associated loss of adenoviral susceptibility and Coxsackie-adenovirus receptor expression in human mesenchymal stem cells. Stem Cells. 22, 1321–1329.

Hussain, F., Morton, P. E., Snippe, M., Sullivan, J., Farmer, C., Martin-Fernandez, M. L.,

Parsons, M. and Santis, G. (2011). CAR Modulates E-Cadherin Dynamics in the Presence of Adenovirus Type 5. PLoS ONE. 6, e23056–11.

Hutchin, M. E., Pickles, R. J. and Yarbrough, W. G. (2000). Efficiency of adenovirus-mediated gene transfer to oropharyngeal epithelial cells correlates with cellular differentiation and human coxsackie and adenovirus receptor expression. Hum. Gene Ther. 11, 2365–2375.

Kallewaard, N. L., Zhang, L., Chen, J.-W., Guttenberg, M., Sanchez, M. D. and Bergelson, J. M. (2009). Tissue-Specific Deletion of the Coxsackievirus and Adenovirus Receptor Protects Mice from Virus-Induced Pancreatitis and Myocarditis. Cell Host & Microbe. 6, 91–98.

Kawabata, K., Sakurai, F., Yamaguchi, T., Hayakawa, T. and Mizuguchi, H. (2005). Efficient gene transfer into mouse embryonic stem cells with adenovirus vectors. Molecular Therapy. 12, 547–554.

Kidder, B. L. and Palmer, S. (2010). Examination of transcriptional networks reveals an important role for TCFAP2C, SMARCA4, and EOMES in trophoblast stem cell maintenance. Genome Research. 20, 458–472.

Kolawole, A. O., Sharma, P., Yan, R., Lewis, K. J. E., Xu, Z., Hostetler, H. A. and Ashbourne Excoffon, K. J. D. (2012). The PDZ1 and PDZ3 Domains of MAGI-1 Regulate the Eight-Exon Isoform of the Coxsackievirus and Adenovirus Receptor. Journal of Virology. 86, 9244–9254.

Krivega, M., Geens, M. and Van de Velde, H. (2014). CAR expression in human embryos and hESC illustrates its role in pluripotency and tight junctions. Reproduction. 148, 531–544.

Kwon, J.-W., Kim, N.-H. and Choi, I. (2016). CXADR is required for AJ and TJ assembly during porcine blastocyst formation. Reproduction. 151, 297–304.

Lacher, M. D., Shiina, M., Chang, P., Keller, D., Tiirikainen, M. I. and Korn, W. M. (2011). ZEB1 limits adenoviral infectability bytranscriptionally repressing the Coxsackievirus and Adenovirus Receptor. Molecular Cancer. 10, 91.

Lacher, M. D., Tiirikainen, M. I., Saunier, E. F., Christian, C., Anders, M., Oft, M., Balmain, A., Akhurst, R. J. and Korn, W. M. (2006). Transforming growth factor-beta receptor inhibition enhances adenoviral infectability of carcinoma cells via up-regulation of Coxsackie and Adenovirus Receptor in conjunction with reversal of epithelial-mesenchymal transition. Cancer Res. 66, 1648–1657.

Leon, R. P., Hedlund, T., Meech, S. J., Li, S., Schaack, J., Hunger, S. P., Duke, R. C. and DeGregori, J. (1998). Adenoviral-mediated gene transfer in lymphocytes. Proc. Natl. Acad. Sci. U.S.A. 95, 13159–13164.

Lim, B.-K., Xiong, D., Dorner, A., Youn, T.-J., Yung, A., Liu, T. I., Gu, Y., Dalton, N. D., Wright, A. T., Evans, S. M., et al. (2008). Coxsackievirus and adenovirus receptor (CAR) mediates atrioventricular-node function and connexin 45 localization in the murine heart. J. Clin. Invest. 118, 2758–2770.

Lisewski, U., Shi, Y., Wrackmeyer, U., Fischer, R., Chen, C., Schirdewan, A., Juttner, R.,

Rathjen, F., Poller, W., Radke, M. H., et al. (2008). The tight junction protein CAR regulates cardiac conduction and cell-cell communication. J Exp Med. 205, 2369–2379.

Liu, J.-Y., Wang, S.-M., Chen, I.-C., Yu, C.-K. and Liu, C.-C. (2013). Hepatic damage caused by coxsackievirus B3 is dependent on age-related tissue tropisms associated with the coxsackievirus-adenovirus receptor. Pathogens Disease. 68, 52–60.

Matthaus, C., Langhorst, H., SchÃ¼tz, L., JÃ¼ttner, R. and Rathjen, F. G. (2017). Cell-cell communication mediated by the CAR subgroup of immunoglobulin cell adhesion molecules in health and disease. Molecular and Cellular Neuroscience. 81, 32–40.

Mirza, M., Hreinsson, J., Strand, M.-L., Hovatta, O., Söder, O., Philipson, L., Pettersson, R. F. and Sollerbrant, K. (2006). Coxsackievirus and adenovirus receptor (CAR) is expressed in male germ cells and forms a complex with the differentiation factor JAM-C in mouse testis. Experimental Cell Research. 312, 817–830.

Mirza, M., Pang, M.-F., Zaini, M. A., Haiko, P., Tammela, T., Alitalo, K., Philipson, L., Fuxe, J. and Sollerbrant, K. (2012). Essential Role of the Coxsackie - and Adenovirus Receptor (CAR) in Development of the Lymphatic System in Mice. PLoS ONE. 7, e37523.

Mirza, M., Petersen, C., Nordqvist, K. and Sollerbrant, K. (2007). Coxsackievirus and Adenovirus Receptor Is Up-Regulated in Migratory Germ Cells during Passage of the Blood-Testis Barrier. Endocrinology. 148, 5459–5469.

Mirza, M., Raschperger, E., Philipson, L., Pettersson, R. F. and Sollerbrant, K. (2005). The cell surface protein coxsackie- and adenovirus receptor (CAR) directly associates with the Ligand-of-Numb Protein-X2 (LNX2). Experimental Cell Research. 309, 110–120.

Morton, P. E., Hicks, A., Nastos, T., Santis, G. and Parsons, M. (2013). CAR regulates epithelial cell junction stability through control of E-cadherin trafficking. Sci. Rep. 3.

Okegawa, T., Pong, R. C., Li, Y., Bergelson, J. M., Sagalowsky, A. I. and Hsieh, J. T. (2001). The mechanism of the growth-inhibitory effect of coxsackie and adenovirus receptor (CAR) on human bladder cancer: a functional analysis of car protein structure. Cancer Res. 61, 6592–6600.

Oh, Y. S., Nah, W. H., Choi, B., Kim, S. H. and Gye, M. C. (2016). Coxsackievirus and Adenovirus Receptor, a Tight Junction Protein, in Peri-Implantation Mouse Embryos. Biology of Reproduction. 95, 5–5.

Patzke, C., Max, K. E. A., Behlke, J., Schreiber, J., Schmidt, H., Dorner, A. A., Kroger, S., Henning, M., Otto, A., Heinemann, U., et al. (2010). The Coxsackievirus-Adenovirus Receptor Reveals Complex Homophilic and Heterophilic Interactions on Neural Cells. Journal of Neuroscience. 30, 2897–2910.

Pazirandeh, A., Sultana, T., Mirza, M., Rozell, B., Hultenby, K., Wallis, K., Vennström, B., Davis, B., Arner, A., Heuchel, R., et al. (2011). Multiple Phenotypes in Adult Mice following Inactivation of the Coxsackievirus and Adenovirus Receptor (Car) Gene. PLoS ONE. 6, e20203.

Raschperger, E., Neve, E. P. A., Wernerson, A., Hultenby, K., Pettersson, R. F. and Majumdar, A. (2008). The coxsackie and adenovirus receptor (CAR) is required for renal epithelial differentiation within the zebrafish pronephros. Developmental Biology. 313, 455–464.

Rebel, V. I., Hartnett, S., Denham, J., Chan, M., Finberg, R. and Sieff, C. A. (2000). Maturation and lineage-specific expression of the coxsackie and adenovirus receptor in hematopoietic cells. Stem Cells. 18, 176–182.

Saito, K., Sakaguchi, M., Iioka, H., Matsui, M., Nakanishi, H., Huh, N. H. and Kondo, E. (2013). Coxsackie and adenovirus receptor is a critical regulator for the survival and growth of oral squamous carcinoma cells. Oncogene. 33, 1274–1286.

Salinas, S., Zussy, C., Loustalot, F., Henaff, D., Menendez, G., Morton, P. E., Parsons, M., Schiavo, G. and Kremer, E. J. (2014). Disruption of the coxsackievirus and adenovirus receptor-homodimeric interaction triggers lipid microdomain- and dynamin-dependent endocytosis and lysosomal targeting. Journal of Biological Chemistry. 289, 680–695.

Shono, A., Tsukaguchi, H., Yaoita, E., Nameta, M., Kurihara, H., Qin, X. S., Yamamoto, T. and Doi, T. (2007). Podocin Participates in the Assembly of Tight Junctions between Foot Processes in Nephrotic Podocytes. Journal of the American Society of Nephrology. 18, 2525–2533.

Sollerbrant, K. (2002). The Coxsackievirus and Adenovirus Receptor (CAR) Forms a Complex with the PDZ Domain-containing Protein Ligand-of-Numb Protein-X (LNX). Journal of Biological Chemistry. 278, 7439–7444.

Su, L., Mruk, D. D. and Cheng, C. Y. (2012). Regulation of the blood-testis barrier by coxsackievirus and adenovirus receptor. Am. J. Physiol., Cell Physiol. 303, C843–53.

Sultana, T., Hou, M., Stukenborg, J. B., Tohonen, V., Inzunza, J., Chagin, A. S. and Sollerbrant, K. (2014). Mice depleted of the coxsackievirus and adenovirus receptor display normal spermatogenesis and an intact blood-testis barrier. Reproduction. 147, 875–883.

Sun, F., Li, Y., Jia, T., Ling, Y., Liang, L., Liu, G., Chen, H. and Chen, S. (2012). Differential expression of coxsackievirus and adenovirus receptor on alveolar epithelial cells between fetal and adult mice determines their different susceptibility to coxsackievirus B infection. Arch Virol. 157, 1101–1111.

Tashiro, K., Hirata, N., Okada, A., Yamaguchi, T., Takayama, K., Mizuguchi, H. and Kawabata, K. (2015). Expression of Coxsackievirus and Adenovirus Receptor Separates Hematopoietic and Cardiac Progenitor Cells in Fetal Liver Kinase 1-Expressing Mesoderm. STEM CELLS Translational Medicine. 4, 424–436.

Tomko, R. P., Johansson, C. B., Totrov, M., Abagyan, R., Frisen, J. and Philipson, L. (2000). Expression of the Adenovirus Receptor and Its Interaction with the Fiber Knob. Experimental Cell Research. 255, 47–55.

Triantafilou, K. and Triantafilou, M. (2004). Lipid-raft-dependent Coxsackievirus B4 internalization and rapid targeting to the Golgi. Virology. 326, 6–19.

Venkatraman, G., Behrens, M., Pyrski, M. and Margolis, F. L. (2005). Expression of Coxsackie-Adenovirus receptor (CAR) in the developing mouse olfactory system. J. Neurocytol. 34, 295–305.

Verdino, P. and Wilson, I. A. (2011). JAML and CAR: Two more players in T-cell activation. Cell Cycle. 10, 1341–1342.

Verdino, P., Witherden, D. A., Ferguson, M. S., Corper, A. L., Schiefner, A., Havran, W. L. and Wilson, I. A. (2011). Molecular Insights into gd T Cell Costimulation by an Anti-JAML Antibody. Structure/Folding and Design. 19, 80–89.

Vincent, T., Neve, E. P. A., Johnson, J. R., Kukalev, A., Rojo, F., Albanell, J., Pietras, K., Virtanen, I., Philipson, L., Leopold, P. L., et al. (2009). A SNAIL1–SMAD3/4 transcriptional repressor complex promotes TGF-β mediated epithelial–mesenchymal transition. Nat Cell Biol. 11, 943–950.

Vincent, T., Pettersson, R. F., Crystal, R. G. and Leopold, P. L. (2004). Cytokine-Mediated Downregulation of Coxsackievirus-Adenovirus Receptor in Endothelial Cells. Journal of Virology. 78, 8047–8058.

Vindrieux, D., Le Corre, L., Hsieh, J. T., Metivier, R., Escobar, P., Caicedo, A., Brigitte, M. and Lazennec, G. (2011). Coxsackie and adenovirus receptor is a target and a mediator of estrogen action in breast cancer. Endocrine Related Cancer. 18, 311–321.

Walters, R. W., Freimuth, P., Moninger, T. O., Ganske, I., Zabner, J. and Welsh, M. J. (2002). Adenovirus fiber disrupts CAR-mediated intercellular adhesion allowing virus escape. Cell. 110, 789–799.

Wang, C. Q. F., Mruk, D. D., Lee, W. M. and Cheng, C. Y. (2007). Coxsackie and adenovirus receptor (CAR) is a product of Sertoli and germ cells in rat testes which is localized at the Sertoli-Sertoli and Sertoli-germ cell interface. Experimental Cell Research. 313, 1373–1392.

Witherden, D. A., Verdino, P., Rieder, S. E., Garijo, O., Mills, R. E., Teyton, L., Fischer, W. H., Wilson, I. A. and Havran, W. L. (2010). The Junctional Adhesion Molecule JAML Is a Costimulatory Receptor for Epithelial T Cell Activation. Science. 329, 1205–1210.

Zen, K., Liu, Y., McCall, I. C., Wu, T., Lee, W., Babbin, B. A., Nusrat, A. and Parkos, C. A. (2005). Neutrophil migration across tight junctions is mediated by adhesive interactions between epithelial coxsackie and adenovirus receptor and a junctional adhesion molecule-like protein on neutrophils. Mol Biol Cell. 16, 2694–2703.
